# Time-lapse sentinel surveillance of SARS-CoV-2 spread in India

**DOI:** 10.1371/journal.pone.0241172

**Published:** 2020-10-22

**Authors:** Mukesh Thakur, Abhishek Singh, Bheem Dutt Joshi, Avijit Ghosh, Sujeet Kumar Singh, Neha Singh, Lalit Kumar Sharma, Kailash Chandra

**Affiliations:** 1 Zoological Survey of India, New Alipore, Kolkata, West Bengal, India; 2 Gujarat Forensic Sciences University, Gandhinagar, Gujarat, India; 3 Smithsonian Conservation Biology Institute, Front Royal, Virginia, United States of America; Universita degli Studi della Campania Luigi Vanvitelli, ITALY

## Abstract

The novel coronavirus 2019 (COVID-19) global pandemic has drastically affected the world economy, raised public anxiety, and placed a substantial psychological burden on the governments and healthcare professionals by affecting over 4.7 million people worldwide. As a preventive measure to minimise the risk of community transmission of severe acute respiratory syndrome coronavirus 2 (SARS-CoV-2) in India, a nationwide lockdown was imposed initially for 21 days to limit the movement of 1.3 billion people. These restrictions continue in most areas, with a conditional relaxation occurring in a few Indian states. In an attempt to assess the emerging mutants of SARS-CoV-2 and determine their spread in India, we analysed 112 complete genomes of SARS-CoV-2 in a time-lapse manner. We found 72 distinct SARS-CoV-2 haplotypes, defined by 143 polymorphic sites and high haplotype diversity, suggesting that this virus possesses a high evolutionary potential. We also demonstrated that early introduction of SARS-CoV-2 into India was from China, Italy and Iran and observed signs of community spread of the virus following its rapid demographic expansion since its first outbreak in the country. Additionally, we identified 18 mutations in the SARS-CoV-2 spike glycoprotein and a few selected mutations showed to increase stability, binding affinity, and molecular flexibility in the overall tertiary structure of the protein that may facilitate interaction between the receptor binding domain (RBD) of spike protein and the human angiotensin-converting enzyme 2 (ACE2) receptor. The study provides a pragmatic view of haplotype-dependent spread of SARS-CoV-2 in India which could be important in tailoring the pharmacologic treatments to be more effective for those infected with the most common haplotypes. The findings based on the time-lapse sentinel surveillance of SARS-CoV-2 will aid in the development of a real-time practical framework to tackle the ongoing, fast-evolving epidemic challenges in the country.

## Introduction

The severe acute respiratory syndrome coronavirus 2 (SARS-CoV-2), emerged in Wuhan, China in early December 2019 [1], stressing local healthcare systems and eventually causing a substantial number of deaths [2]. The virus spread rapidly after the outbreak, and as of 19 May 2020, it has caused 311 847 deaths worldwide (COVID-19 Situation Report, 119). SARS-CoV-2, being zoonotic, originated in bats [3] and may have been transmitted to humans through pangolins as an intermediate host [1]. Although SARS-CoV-2 spreads rapidly regardless of nationality, a recent study suggests that East Asian human populations may be more susceptible to infection due to a relatively higher angiotensin-converting enzyme 2 (ACE2) receptor expression in various tissues [4]. This receptor is thought to be crucial for viral entry into human cells through its interaction with SARS-CoV-2 spike glycoproteins [4]. While many researchers have conducted, and are currently undertaking, experiments to impede this interaction as a viable therapy, the highly mutagenic nature of SARS-CoV-2 may make this a difficult task. Indeed, SARS-CoV-2 has been reported to accumulate an average of about one to two mutations per month [5]. The accumulation of viral mutations may affect virulence significantly, and raises concern about the development of a broad-spectrum vaccine to combat this pandemic [6, 7]. Thus, it is essential to identify the mutations in the key viral proteins (e.g. the spike protein) and track the spread of the SARS-CoV-2 haplotypes that harbour these mutations [5, 8].

In addition to investigate the emerging mutations in SARS-CoV-2, scientists across the globe have started analysing viral genomes to address its evolutionary history [9], phylogenetic relationships [10, 11] and entry routes into different countries [12, 13]. To facilitate this effort, the Global Initiative on Sharing Avian Influenza Data (GISAID; https://www.gisaid.org/), developed a dedicated coronavirus repository in December 2019. Furthermore, Nextstrain, the open-source pathogen genome project, with data plugged-in from GISAID, demonstrates a global panoramic view of the SARS-CoV-2 pandemic. It includes SARS-CoV-2 phylogenetic analyses, along with epidemiological surveillance data and information on the movement of the virus from China to other parts of the globe [14].

In India, most of the early COVID-19 cases are believed to have originated from foreign travellers rather than being transmitted within the country. The first three cases in India were identified from 30 January to 3 February 2020, in Kerala, with a travel history related to Wuhan, China [15]. On 2 March 2020, the Union Health Ministry reported two more cases: a 45-year-old man in Delhi who had travelled back from Italy and a 24-year-old engineer in Hyderabad who recently returned from Dubai. Also, a group of 23 Italian tourists arrived in Delhi on 21 February 2020 and travelled to Rajasthan. Sixteen of these tourists and their Indian driver tested positive for COVID-19 on 4 March 2020 before they came into contact with at least 215 people across six districts of Rajasthan. Subsequently, on 7 March 2020, the Government of India brought more than 100 swab samples from Indian pilgrims in Iran that were suspected to be infected by SARS-CoV-2, a majority of which belonged to the Union Territory of Ladakh who visited Iran for a religious festival, *Ziarat*. Eventually, many cases of COVID-19 emerged in different parts of the country with an official record of 101 138 total cases and 3163 deaths from the 33 states/union territories of India as of 19 May 2020, 08:00 GMT+5:30 (https://www.mygov.in/covid-19/?cbps=1).

In this study, we assessed 112 complete SARS-CoV-2 viral genomes, including those from the viral isolates of the cases identified above, to track the origins of different viral haplotypes and their associated spread in Indian communities. Moreover, from the identified haplotypes, we investigated the various mutations in the spike glycoprotein. A total of 72 SARS-CoV-2 haplotypes were identified from the analysed genomes, whose spread varied within the Indian population. Moreover, the viral genomes we assessed contained 18 spike protein mutations that variably affected spike-ACE2 receptor interactions.

## Materials and methods

### Data mining

We downloaded 112 genome sequences of SARS-CoV-2 submitted from India on the GISAID database until 15 May 2020 (S1 Table in S1 File). These sequences also included the first two cases of SARS-CoV-2 (EPI_ISL_413522–23) diagnosed in Kerala, the first case observed in Hyderabad (EPI_ISL_431101), the first five Italian tourists that travelled to Rajasthan and their Indian driver (EPI_ISL_420543, 420545, 420547, 420549, 420553, and 420551), and 14 Indian pilgrims from the Union Territory of Ladakh that were sampled in Iran (EPI_ISL_421662–72, EPI_ISL_424361–63). Additionally, we included the first Wuhan 1 reference sequence, (henceforth, Wuhan-Hu-1; GenBank ID-MN908947.3), three of the first 16 cases observed in Italy (EPI_ISL_417445–47), and a reference genome from Iran (EPI_ISL_424349) to geographically assign the first few cases that appeared in India. We aligned all sequences using MUSCLE v3.8.31 [16], with reference to Wuhan-Hu-1.

### Genetic diversity and phylogenetic network analyses

We estimated genetic diversity, *i*.*e*. polymorphic sites (P), haplotypes (H), the mean number of pairwise nucleotide differences (K), and haplotype (Hd) and nucleotide diversity (*π*), using DnaSP v6.12 [17]. For median-joining network analysis, we classified genomes into three time frames, *i*.*e*. T1−35 genomes (early cases of virus outbreaks in India and available as of 21 April 2020), T2−53 genomes (available as of 2 May 2020), and T3−112 genomes available as of 5 May 2020. The median-joining network [18] was obtained using NETWORK v4.5.1.0 (www.fluxus-engineering.com). Network calculations were carried out by assigning equal weights to all variable sites, with default values for the epsilon parameter (epsilon = 0) to minimise alternative median networks.

### Demography assessment

Parameters like the mismatch distribution test, Harpending's raggedness index (Rg), and the sum-of-squared deviations (SSD) were calculated to test for demographic expansion under the sudden expansion model using Arlequin v3.5 [19]. Neutrality tests were conducted based on the infinite sites model of Tajima’s D [20] and Fu’s Fs [21], using DnaSP v6.12 to evaluate demographic effects [19]. To date the changes in the effective population size over time, we used Bayesian Skyline Plot (BSP) analysis as implemented in BEAST v.2.5 [22], with 8 × 10^−4^ subs per site per year as the substitution rate [11, 23]. Markov chains were run for 2.5 × 10^7^ generations and sampled every 1000 generations, with the first 2500 samples discarded as burn-in. Other parameters were set as default values, and the output was visualised in TRACER v.1.6 [24]. In addition, we also retrieved real-time data of COVID-19 cases in India as of 26 April 2020 from the Ministry of Health and Family Welfare (MoHFW; https://www.mohfw.gov.in/index.php) and transformed it into log10 format to match the virus growth and demography patterns from our BSP analysis.

### Mapping mutations and modelling three dimensional structures

We mapped mutations in the spike glycoprotein with respect to the reference protein of the genome Wuhan-Hu-1 (GenBank accession MN908947.3), using a dedicated Coronavirus Typing tool of Genome Detective v.1.13 [25]. We also estimated mutation frequency in viral genomes at three time interval as a ratio of the number of non-synonymous mutations observed in the spike protein to the total number of genomes analysed. Further, we prioritised five mutants (M1 to M5) for three-dimensional structure modelling as three mutants- M1, M2 and M3 carried mutations in the receptor binding domain (RBD) (RBD site: 336–516 aa), while M4 carried a mutation in the S1 domain of the spike protein (S1: 515–667 aa) and was the most abundant in the Indian viral genomes. Mutant M5 carried a mutation in the S1 (S1: 515–667 aa) and S2 domain (S2:668–1273 aa) of the spike protein, which occurred in a haplotype H_40 that spread widely in seven Indian states. We modelled the three-dimensional structure of these selected mutants and the wild-type protein (Wuhan-Hu-1 protein; GenBank accession MN908947.3) using the Phyre2 protein fold recognition server [26] and further refined using the 3D refine protein structure refinement server [27]. The overall quality of the modelled structures was assessed using the RAMPAGE server [28]. We then employed a root mean square deviation (RMSD) analysis for a quantitative assessment of the similarity between mutant and wild-type proteins using the TM-score server [29].

### Molecular docking with the human ACE2 receptor

We retrieved a three-dimensional structure of the human ACE2 receptor from the Protein Data Bank (accession ID: 6LZG). We performed molecular docking using the HADDOCK 2.4 webserver [30] to assess the interaction pattern of mutated and wild-type spike glycoproteins with the ACE2 receptor. We visualised the interaction pattern using Discovery Studio 2020 [31] and analysed the binding affinity of docked structures using the PRODIGY webserver [32]. The amino acid residues involved in the interaction between the spike glycoprotein and human ACE2 receptor were identified using the PDBePISA server [33].

### Effects of selected mutations on protein dynamics

We analysed the effects of the aforementioned mutations on the stability and the molecular flexibility of the tertiary structure of the spike protein by determining the free energy difference (ΔΔG) and vibrational entropy energy (ΔΔSVib ENCoM). This was performed using the mutation effects prediction tool on the DynaMut web server [34]. In addition, we also assessed the effect of these mutations on deformation energies and the interactions of these residues with neighbouring amino acids.

## Results

### Genetic polymorphism and phylogenetic network analysis

In assessing 112 SARS-CoV-2 genomes from India, we obtained 72 distinct haplotypes, defined by 143 polymorphic sites, an average nucleotide difference of 6.9, with 0.963 Hd and 0.00027 *π* (S2 Table in S1 File). The high haplotype diversity with low nucleotide diversity suggests a recent divergence and high evolutionary potential for SARS-CoV-2.

#### T_1_: 35 SARS-CoV-2 genomes

**Fig 1:**
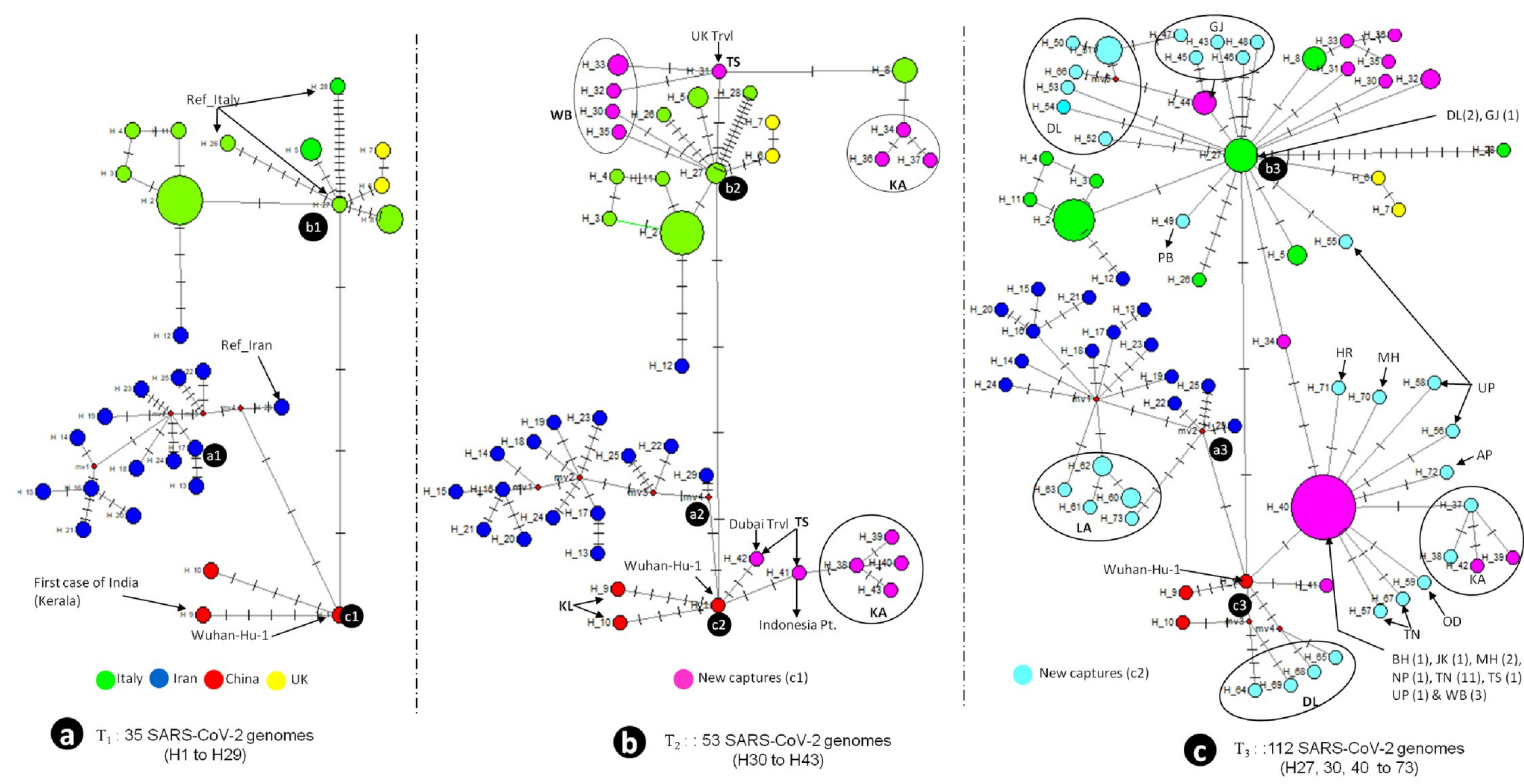
Phylogenetic network analysis of complete SARS-CoV-2 genomes identified in India. **(a).** Analysis based on 35 complete genomes available on the 21st of April 2020. **(b).** Analysis based on 53 complete genomes available on the 2nd of May 2020. **(c)** Analysis based on 112 complete genomes available on the 5th of May 2020 and retrieved from GISAID database.

Thirty-five coronavirus genomes available as of 21 April 2020, yielded 24 haplotypes, grouped into three major clusters—a1, b1, and c1 (Fig 1A). Cluster a1 included 14 haplotypes (58%; occurred in the 14 early cases of Indian pilgrims sampled from Iran) and were assigned with the reference genome of Iran (H_29). Cluster b1 included six haplotypes (25%; occurred in 15 early cases that appeared in India), and were assigned with the first three cases from Italy (H_26 to H_28). In clade b1, haplotype H_2 was the most prominent and was shared by the five Italian tourists that visited India and diagnosed with COVID-19 in Rajasthan. Additionally, cluster c1 included only two haplotypes, H_9 and H_10 (first coronavirus cases diagnosed in Kerala), and evolved from the Wuhan-Hu-1 (H_1), by means of five mutations. Thus, the early cases reported from India were genetically assigned following the patients' travel history from China, Iran, and Italy (Fig 1A).

#### T_2_: 53 SARS-CoV-2 genomes

The inclusion of 18 new genomes from the previous time frame as of 2 May 2020, yielded 14 new haplotypes distributed in three States *i*.*e*. four in West Bengal, seven in Karnataka, and three in Telangana (Fig 1B; all new haplotypes are shown in pink). The clades b2 and c2 which originated from Italy and China proliferated further in India. All haplotypes in West Bengal, H_30, 32, 33, and 35, and haplotypes H_34, 36, and 37 in Karnataka, whose associated patients had no foreign travel history, evolved from a single mutation in the haplotypes H_31 and H_8, respectively, and clustered within clade b2. While the other four haplotypes, H_38, 39, 40, and 43 in Karnataka, evolved from haplotype H_41, and was transmitted from an Indonesian citizen diagnosed in Telangana State. Moreover, in Telangana, we obtained haplotypes having mixed origin, with virus having travelled from the United Kingdom (H_31; clade b2 in Fig 1B) and Dubai (H_42; clade c2 in Fig 1B). In this time frame, there was no further capture of any haplotype in clade a2, originating from Iran.

#### T_3_: 112 SARS-CoV-2 genomes

Analysing the 112 SARS-CoV-2 genomes, available as of 5 May 2020, yielded a total of 72 haplotypes, 34 of which were unique from those obtained earlier (Fig 1C; all new haplotypes are shown in cyan). Interestingly, two haplotypes, H_27 in clade b3 and H_40 in clade a3 (which evolved by a single mutation in Wuhan-Hu-1), spread widely in India forming star-like phylogenies. This suggests that there was community-mediated spread of the coronavirus, since most of the patients did not have any travel history abroad. Haplotypes observed in Delhi also had mixed ancestry, with clade b3 (H_27, 50, 51–54, and 66) and c3 (H_64, 65, 68, and 69) originating from Italy and China, respectively. Five new haplotypes, H_60–63 and 73, belonged to the pilgrims from the Union Territory of Ladakh clustered with the a3 clade (Fig 1C).

### Demography of SARS-CoV-2 in India

**Fig 2:**
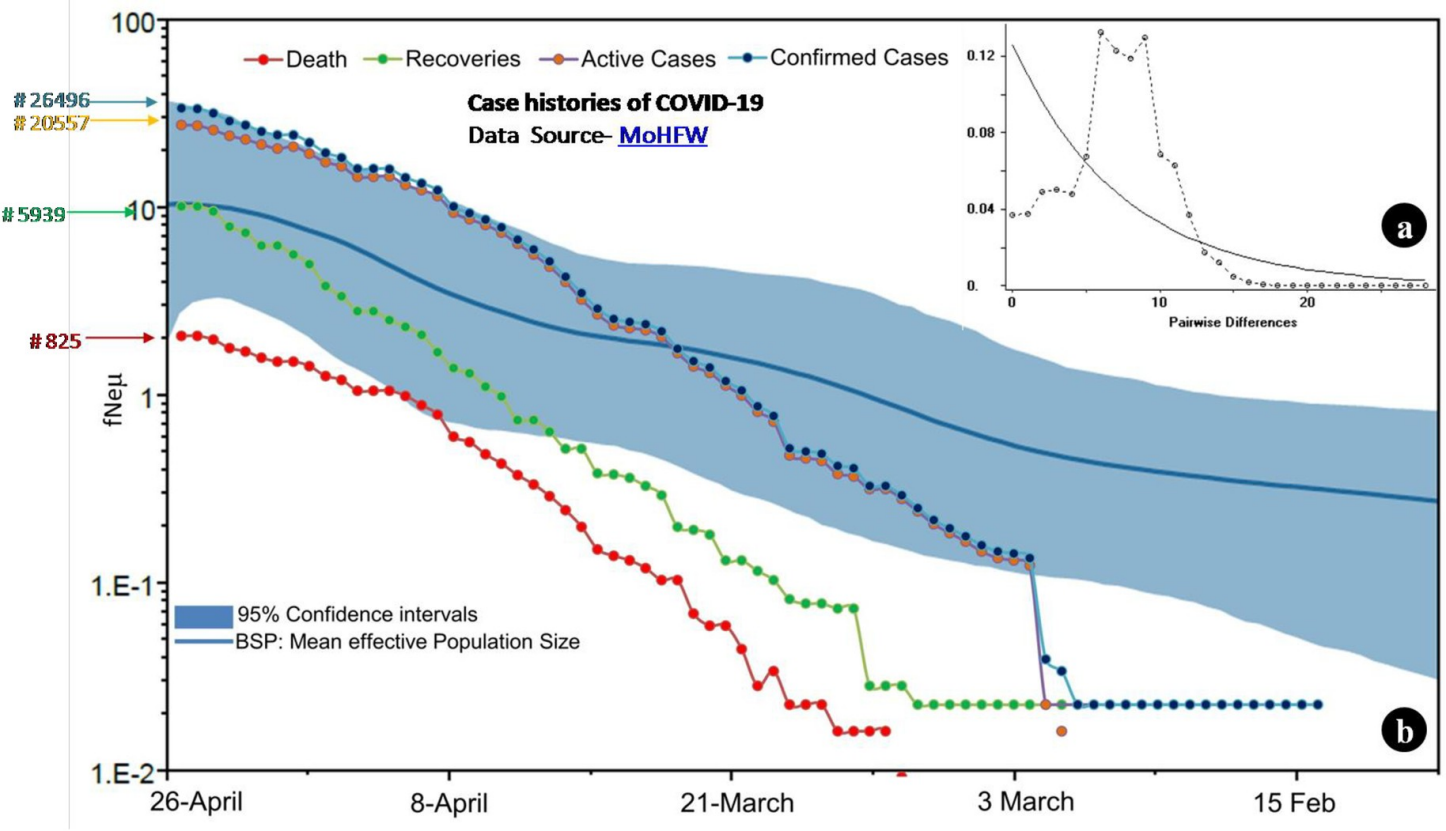
**(a)** Mismatch distribution curve of pairwise differences among SARS-CoV-2 genomes. **(b)** Demographic history of SARS-CoV-2 genomes estimated using Coalescent Bayesian Skyline plot. We applied a strict molecular clock and a substitution rate at 8×10^−4^/site/year to the MCMC run for 2.5×10^7^ generations, sampled at every 1000 generations and discarded the first 2500 samples as burn-in. Other parameters were set as default values and results are visualized using TRACER v. 1.6. Bayesian skyline plot shows an overall increasing population size. The solid line represents the median estimates of *Neμ* (Ne = effective population size; μ = generation time) and the blue lines around median estimates indicate the 95% highest posterior density (HPD) estimate of the historic effective population size. The lines with nodes overlaid on BSP represented case histories of COVID-19 with data retrieved from MoHFW. The data was available in absolute number before transforming it to the log form to best fit with the axis also represented BSP results. The red line depicted number of deaths, green- number of recoveries, pink- number of active cases and blue represented total number of confirmed cases.

We obtained a bimodal mismatch distribution curve (Fig 2A) that supports the larger mismatch values over time as mutations accumulate in the population and possibly indicates a stable demography [35]. The estimate of Tajima's D (-2.44849; P < 0.01) and Fu's Fs (-21.17755; P < 0.006) calculated based on the infinite sites model of mutation were significant and suggested that the population has experienced a recent expansion. The mismatch distribution curve of pairwise genetic differences fit the sudden expansion model in effective population size (SSD 0.00180, P = 0.9). Further, a bimodal mismatch distribution with a non-significant raggedness index (Rg 0.00425, P = 0.87), suggests a recent population expansion and possible demographic stability (S2 Table in S1 File). Taken together, the mismatch distribution curve that possibly depicted a situation of general demographic stability differed from the estimates of Tajima's D, Fu's Fs, SSD, and Rg, which favoured a recent demographical expansion. The BSP results showed an overall increasing population with a rapid expansion in the effective population size since the first outbreak of COVID-19 in the country. This fit well with the COVID-19 case statistics of India (Fig 2B; MoHFW data overlaid with BSP results).

### Mutations in the spike glycoprotein and structural variants

We obtained 18 SARS-CoV-2 mutants that carried mutations in their spike glycoproteins, *i*.*e*. six in the S1: 1–335 region; three in the RBD: 336–516 site; two in the S1: 515–667 region, and seven in the S2: 668–1273 region (S2 Table in S1 File). The overall mutation frequency observed in three time frames ranged from 0.44–0.64 with lowest estimates observed at T2 (0.44) in comparison to T1 (0.62) and T3 frames (0.64) (S3 Table in S1 File). Among all the observed mutations, the mutation D614G was dominant and most occurred mutation at all three time frames with observed frequency ranged from 0.32–0.46. Furthermore, majority of mutations observed at T1 and T2 were relatively present in high frequencies than the mutations observed at T3 (S4 Table in S1 File).

Among the prioritised five mutants (M1 to M5), the three mutants that carried mutations in RBD site were M1 (S438F (22875C>T) + D614G (23403A>G)) in EPI_ISL_420543 and EPI_ISL_420547 (H_4 and H_11 from Italian tourists), M2 (R408I (22785G>T)) in EPI_ISL_413522 (H_9 from Kerala -the first case in India), and M3 (I434K (22863T>A) + D614G (23403A>G)) in EPI_ISL_424362 (H_12 from an Indian sampled in Iran). Interestingly, mutant M4, carrying mutation D614G (23403A>G), was the most abundant mutation present in the analysed genomes. In contrast, mutant M5, carrying mutations K77M (21792A>T) and A771V (23874C>T), was present in the most widely spread haplotype, H_40 (found in 21 genomes that spread amongst seven Indian states; Fig 1C).

The Ramachandran plot analysis of the five mutant modelled proteins revealed more than 93% of the residues were in favoured regions and less than 1.5% were outliers (S7 Table in S1 File). Since a single amino acid change can affect the overall structure of a protein, we performed a quantitative assessment of the similarity between the wild-type and mutant models. However, this analysis revealed no significant structural differences between wild-type and mutant proteins, as the TM-score for all the mutant proteins were near to one (S8 Table in S1 File).

### Molecular docking of selected mutants with the ACE2 receptor

Based on the lowest Z score, we selected best fit docked structures for each mutant which yielded variable binding affinities with the ACE2 receptor (S1A–S1E Fig in S1 File). Interestingly, mutants M4 and M5 of the spike glycoprotein, whose mutations were present outside the RBD site, exhibited increased binding affinities (-15.4 and -14.2 kcal/mol, respectively) as compared to the wild-type protein (-13.2 kcal/mol). Alternatively, mutants M1, M2, and M3, whose mutations were within the RBD site, exhibited relatively low binding affinities (-12.7, -12.7, and -13 kcal/mol) as compared to the wild-type protein (S6 Table in S1 File). Molecular docking between the RBD site of the wild-type, M4, and M5 spike proteins with the ACE2 receptor revealed a lower number of residues involved in salt bridge interactions in comparison to the M1, M2, and M3 mutant proteins (S9 Table in S1 File). Binding domain analysis of three RBD site mutants revealed no direct involvement of mutations S438F, R408I, and I434K in the interaction between the RBD and the ACE2 receptor (S1A–S1E Fig in S1 File).

### Mutation effects on protein dynamics

**Fig 3:**
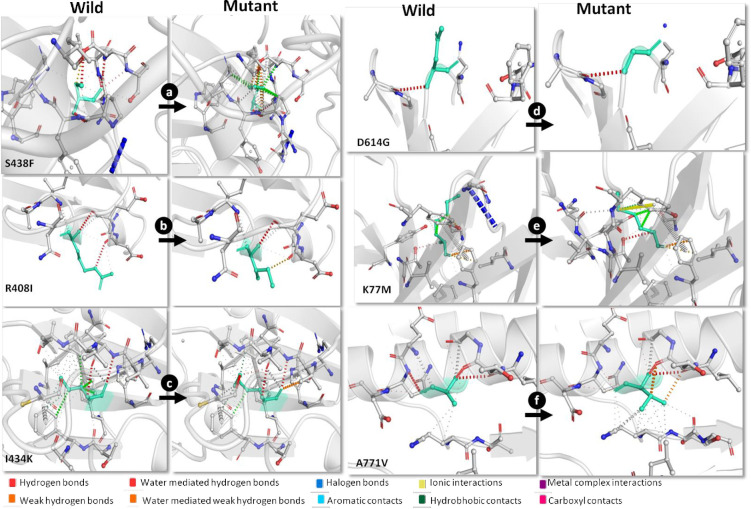
Interaction pattern of wild and mutant residue with the surrounding residues of protein. Wild-type and mutant residues are colored in light-green.

We found ΔΔG, higher than zero for all mutations except I434K, indicating that these alterations had a stabilizing effect (S10 Table in S1 File). The ΔΔSVib ENCoM revealed that three mutations i.e. S438F, K77M, and A771V decreased the molecular flexibility of the tertiary spike protein structure, whereas mutations R408I, I434K, and D614G increased its flexibility (S10 Table in S1 File). Interestingly, mutation D614G increased the molecular flexibility of the overall tertiary structure of the protein including the RBD site, whereas mutation I434K increased molecular flexibility largely at the RBD site (S2C and S2D Fig in S1 File). The visual analysis of deformation energies revealed that the mutations mediated changes in the structural conformations at three prominent positions including one at the RBD site and two in the S1 subunit of the tertiary structure of the spike protein (S3–S5 Figs in S1 File). An investigation into the intramolecular interactions revealed that the S438F mutation containing phenylalanine showed breakage of hydrogen bonds and the formation of a few hydrophobic contacts, which might affect the correct folding of the protein (Fig 3A). Alternatively, the R408I mutation resulted in the loss of hydrogen bonds and the formation of ionic bonds. Moreover, the I434K mutation changed the hydrophobic interactions within the protein (Fig 3B and 3C). Further, the D614G mutation, which has an aspartic acid changing to a glycine, introduces a neutral residue in place of a negatively charge residue (Fig 3D). The mutated glycine residue located on the surface of the domain is one of the most flexible residues and may possibly change the rigidity of the protein; however, we did not observe any such changes in the intramolecular interactions. The other two mutations K77M and A771V showed some changes in the intramolecular interaction pattern, with K77M causing loss of halogen bonds and the formation of an ionic contact. The A771V mutation formed new ionic interactions and showed weak hydrogen bonding (Fig 3E and 3F).

## Discussion

SARS-CoV-2 is a highly mutagenic virus, as is evident by observing the numerous mutations, high haplotype diversity, and its reported potential to evolve rapidly under different climatic conditions [36]. In this study, we analysed 112 high-quality SARS-CoV-2 genomes in a time-lapse analysis and demonstrated that the early introduction of the coronavirus in India was related to travel through China, Italy, and Iran (Fig 1A). The H_9 and H_10 haplotypes, which originated from Wuhan-Hu-1 through five mutations, were the first cases in India, and were officially diagnosed on 30 January 2020 [15]. After diagnosing these cases, approximately 3400 people that came into contact with them were put under quarantine to contain the outbreak. However, no formal record exists regarding how many of them eventually developed coronavirus infections. The network tree being monophyletic provides pragmatic evidence that Wuhan, China was the first source of SARS-CoV-2 in India. Subsequently, the virus invaded India from Italy and Iran, and possibly from various other European countries.

Community spread of SARs-CoV-2 is a significant concern in India, as with many other nations, as that is the most likely mechanism for widespread infections. After analysing 53 SARS-CoV-2 genomes in the second time frame, we obtained two viral clades that originated in Italy and China and proliferated further in India; possibly indicating some early signs of community spread. For example, in Karnataka, the coronavirus spread from patients having haplotype H_8 in clade b2 and H_41 in clade c2 (Fig 1B). Based on the known history of H_8, the patient infected with this haplotype came into contact with another patient who travelled from Italy. Moreover, H_41, which was from an Indonesian citizen who tested positive for SARS-CoV-2 in Telangana State, transmitted infection to an individual harbouring haplotype H_38, who then further spread the infection to other people (H_39, 40, and 43). Interestingly, haplotype H_42 in clade c2, which evolved through three mutations directly from Wuhan-Hu-1 (Fig 1B), belonged to an early case reported in Hyderabad from a boy who returned from Dubai, also indicating that the virus travelled to the United Arab Emirates from China.

Of the 112 genomes analysed, we found that haplotypes H_27 in clade b3 and H_40 in clade c3 showed star-like phylogenetic networks. This indicated a substantial community spread of the virus, as most of these patients did not have any travel history abroad. After tracing the travel patterns of patients with haplotype H_27 and H_40, we found that H_27 was distributed to Delhi, Gujarat, Punjab, and Uttar Pradesh. H_40 spread even more widely, with contacts occurring in Haryana, Maharashtra, Uttar Pradesh, Andhra Pradesh, Karnataka, Odisha, Bihar, Jammu and Kashmir, Tamilnadu, Telangana, West Bengal, and Nepal. Since most of the newly captured haplotypes in the third time frame evolved during the national lockdown in India, this suggests that there was a significant local spread of the virus and that the virus had a high evolutionary rate. In our phylogenetic network analysis, we obtained two dominant ‘b’ and ‘c’ superclades (Fig 1A–1C), which is in accordance with Mavian et al. [14] that found superclade ‘A, also known as the ‘European clade’ (equal to clade ‘b’ in the present study) and superclade ‘B, also known as the ‘East Asian clade’ (equal to clade ‘c’ in the present study) based on viral origins.

The neutrality tests supported that SARS-CoV-2 in India has experienced a recent expansion along with low nucleotide and high haplotype diversity [37]. Further, coronavirus cases have been increasing in India since its outbreak in late January 2020. The MoHFW data overlaid with the BSP results revealed 26 496 confirmed cases in India as of 26 April 2020 (Fig 2B), and this rose to 58 802 active cases as of 19 May 2020 (https://www.mygov.in/covid-19/?cbps=1). This validated the BSP results and demonstrated a rapid expansion of SARS-CoV-2 in India. Hence, we interpret the bimodal curve of mismatch distribution as a result of sub-structuring as various haplotypes of diverged genetic lineages arrived in India. The differential selection of newly emerged mutants could be another possible reason for this demographic scenario.

Mutation mapping of the analysed genomes revealed 18 mutations in the spike glycoprotein, which itself is an indicator of the high evolutionary potential of SARS-CoV-2. Among the five selected mutants, M4 and M5 carried mutations outside the RBD site. However, these mutations failed to elicit any significant structural changes when compared to the wild-type protein. Surprisingly, M4 and M5 exhibited an increased cryptic binding affinity for the ACE2 receptor (S6 Table in S1 File). Further, analysis of the molecular interactions between the RBD site of the wild-type, M4, and M5 spike proteins with the human ACE2 receptor revealed a lower number of residues involved in salt bridge interactions as compared to the mutant proteins, M1, M2, and M3 (S7 Table in S1 File). Binding domain analysis of the three RBD site mutants revealed no direct involvement of mutations S438F, R408I, and I434K in the interaction between RBD and the ACE2 receptor (S1A–S1E Fig in S1 File).

A few studies raised concern by demonstrating differential transmission potential of virus clades. For example, S type strain of the coronavirus was reported to have less transmission as accounted for only 3.7% of the viral isolates in Wuhan, China compared to 96.3% for the L type strain. However, L type strain outside of Wuhan showed 61.3% and S type strain showed 38.4% viral isolates [5]. These differences of viral isolates though provided with limited dataset and evidences that lacked differential virulence of a phylogenetic clade [5, 8, 38]. We recorded haplotypes H_27 and H_40 proliferated widely in the country, relatively in high frequency than others, however, we also did not draw a direct link whether or not, the widespread distribution of these haplotypes was a result of differential virulence or just caught by chance.

The D614G mutation in the spike protein was the most common mutation in the Indian SARS-CoV-2 genomes, and was also prevalent in the viral isolates from Europe [11] and North America [39]. The mutation frequency analysis also suggested that the mutation D614G was the most frequent and prevalent mutation throughout the time period. This mutation increased the molecular flexibility of the overall tertiary structure of the protein including the RBD site. Furthermore, this mutation introduces the most flexible residue, glycine, which changed the overall rigidity of the protein. Thus, we speculate that changes in the intramolecular interaction patterns caused by mutations in the RBD site and more specifically by the mutation of D614G (occurred outside the RBD site), were plausibly responsible for the increased stability and binding affinity of RBD with the ACE2 receptor. In this context, Li et al. [40] provided a new direction by demonstrating that different ACE2 variants could reduce the association between ACE2 and S-protein in the case of SARS-CoV and, therefore, the expression level and pattern of human ACE2 expression in different tissues is another area of research that needs to look into for understanding susceptibility, symptoms, and outcome of SARS-CoV-2 infection.

Given the genetic diversity of SARS-CoV-2 identified in this and other studies, researchers and healthcare professionals face a daunting challenge in combating the spread of COVID-19. Indeed, the information generated in this study through time-lapse sentinel surveillance of SARS-CoV-2 in India illustrates its high mutagenic capacity and its effect on virus spread within a community. We recommend that clinics aid in tracking viral genotypes to help improve our understanding of strain or mutant biased virulence.

## Supporting information

S1 File (DOC)

## References

[1] Zhang YZ, Holmes EC. A Genomic Perspective on the Origin and Emergence of SARS-CoV-2. Cell. 2020; 181: (2) 223–227. 10.1016/j.cell.2020.03.035 32220310 PMC7194821

[2] Ferretti L, Wymant C, Kendall M, Zhao L, Nurtay A, Bonsall D, et al Quantifying dynamics of SARS-CoV-2 transmission suggests that epidemic control and avoidance is feasible through instantaneous digital contact tracing. Science. 2020; 368: (6491) eabb6936 10.1126/science.abb6936 32234805 PMC7164555

[3] Zhou P, Yang XL, Wang XG, Hu B, Zhang L, Zhang W, et al A pneumonia outbreak associated with a new coronavirus of probable bat origin. Nature. 2020; 579:270–273. 10.1038/s41586-020-2012-7 32015507 PMC7095418

[4] Cao Y, Li L, Feng Z, Wan S, Huang P, Sun X, et al Comparative genetic analysis of the novel coronavirus (2019-nCoV/SARS-CoV-2) receptor ACE2 in different populations. Cell Discovery. 2020; 6:11 10.1038/s41421-020-0147-1 32133153 PMC7040011

[5] Tang X, Wu C, Li X, Song Y, Yao X, Wu X, et al On the origin and continuing evolution of SARS-CoV-2. Natl Sci Rev. 2020; nwaa036. 10.1093/nsr/nwaa036 PMC7107875 34676127

[6] Jia Y, Shen G, Zhang Y, Huang KS, Ho HY, Hor WS. Analysis of the mutation dynamics of SARS-CoV-2 reveals the spread history and emergence of RBD mutant with lower ACE2 binding affinity. bioRxiv. 2020; 04.09.034942.

[7] Pachetti M, Marini B, Benedetti F, Giudici F, Mauro E, Storici P, et al Emerging SARS-CoV-2 mutation hot spots include a novel RNA-dependent-RNA polymerase variant. J Transl Med. 2020; 18: 179 10.1186/s12967-020-02344-6 32321524 PMC7174922

[8] Sanche S, Lin YT, Xu C, Romero-Severson E, Hengartner N, Ke R. High Contagiousness and Rapid Spread of Severe Acute Respiratory Syndrome Coronavirus 2. Emerg Infect Dis J. 2020; 26(7). 10.3201/eid2607.200282 PMC7323562 32255761

[9] Li Q, Guan X, Wu P, Wang X, Zhou L, Tong Y, et al Early Transmission Dynamics in Wuhan, China, of Novel Coronavirus–Infected Pneumonia. N Engl J Med. 2020; 382 (13): 1199–1207. 10.1056/NEJMoa2001316 31995857 PMC7121484

[10] Forster P, Forster L, Renfrew C, Forster M, et al Phylogenetic network analysis of SARS-CoV-2 genomes. Proc Natl Acad Sci U S A. 2020; 117 (17): 9241–9243. 10.1073/pnas.2004999117 32269081 PMC7196762

[11] Zehender G, Lai A, Bergna A, Meroni L, Riva A, Balotta C, et al Genomic characterisation and phylogenetic analysis of SARS‐COV‐2 in Italy. J Med Virol. 2020; 1–4. 10.1002/jmv.25577 32222993 PMC7228393

[12] Eden JS, Rockett R, Carter I, Rahman H, Ligt JD, Hadfield J, et al An emergent clade of SARS-CoV-2 linked to returned travellers from Iran. Virus Evolution. 2020; 6 (1): veaa027 10.1093/ve/veaa027 32296544 PMC7147362

[13] Matsuda T, Suzuki H, Ogata N. Phylogenetic analyses of the severe acute respiratory syndrome coronavirus 2 reflected the several routes of introduction to Taiwan, the United States, and Japan. arXiv. 2020; 2002.08802v2.

[14] Mavian C, Marini S, Prosperi M, Salemi M. A snapshot of SARS-CoV-2 genome availability up to March 30th, 2020 and its implications. BioRxiv. 2020; 04.01.020594.

[15] Yadav PD, Potdar VA, Choudhary ML, Nyayanit DA, Agrawal M, Jadhav SM, et al Full-genome sequences of the first two SARS-CoV-2 viruses from India. Indian J Med Res. 2020; 151 (2) 200–209. 32242873 10.4103/ijmr.IJMR_663_20 PMC7258756

[16] Edgar RC. MUSCLE: Multiple Sequence Alignment with High Accuracy and High Throughput. Nucleic Acids Res. 2004; 32 (5): 1792–7. 10.1093/nar/gkh340 15034147 PMC390337

[17] Rozas J, Ferrer-Mata A, Sanchez-DelBarrio JC, Guirao-Rico S, Librado P, Ramos-Onsins SE, et al DnaSP 6: DNA Sequence Polymorphism Analysis of Large Datasets. Mol Biol Evol. 2017; 34 (12): 3299–3302. 10.1093/molbev/msx248 29029172

[18] Bandelt HJ, Forster P, Röhl A. Median-joining networks for inferring intraspecific phylogenies. Mol Biol Evol. 1999; 16 (1):37–48. 10.1093/oxfordjournals.molbev.a026036 10331250

[19] Excoffier L, Lischer HE. ARLEQUIN suite ver 3.5: a new series of programs to perform population genetics analyses under Linux and Windows. Mol Ecol Resour. 2010; 10 (3): 564–7. 10.1111/j.1755-0998.2010.02847.x 21565059

[20] Tajima F. Statistical method for testing the neutral mutation hypothesis by DNA polymorphism. Genetics. 1989; 123 (3): 585–595. 2513255 10.1093/genetics/123.3.585 PMC1203831

[21] Fu YX. Statistical tests of neutrality of mutations against population growth, hitchhiking and background selection. Genetics. 1997; 147 (2): 915–925. 9335623 10.1093/genetics/147.2.915 PMC1208208

[22] Bouckaert R, Vaughan TG, Barido-Sottani J, Duchêne S, Fourment M, Gavryushkina A, et al BEAST 2.5: An advanced software platform for Bayesian evolutionary analysis. PLoS Comput Biol. 2019; 15 (4): e1006650 10.1371/journal.pcbi.1006650 30958812 PMC6472827

[23] Lai A, Bergna A, Acciarri C, Galli M, Zehender G. Early phylogenetic estimate of the effective reproduction number of SARS‐CoV‐2. J Med Virol. 2020; 92: 675–679 10.1002/jmv.25723 32096566 PMC7228357

[24] Rambaut A, Drummond AJ, Xie D, Baele G, Suchard MA, et al Posterior summarisation in Bayesian phylogenetics using Tracer 1.7. Syst Biol. 2018; 67: (5) 901–904. 10.1093/sysbio/syy032 29718447 PMC6101584

[25] Cleemput S, Dumon W, Fonseca V, Karim WA, Giovanetti M, Alcantara LC, et al Genome Detective Coronavirus Typing Tool for rapid identification and characterization of novel coronavirus genomes. Bioinformatics. 2020; btaa145. 10.1093/bioinformatics/btaa145 PMC7112083 32108862

[26] Kelley LA, Mezulis S, Yates CM, Wass MN, Sternberg MJE. (2015). The Phyre2 Web Portal for Protein Modeling, Prediction and Analysis. Nature protocols. 2015; 10: 845–58. 10.1038/nprot.2015.053 25950237 PMC5298202

[27] Bhattacharya D, Nowotny J, Cao R, Cheng J. 3Drefine: an interactive web server for efficient protein structure refinement. Nucleic Acids Res. 2016; 44(W1):W406–9. 10.1093/nar/gkw336 27131371 PMC4987902

[28] Lovell SC, Davis IW, Arendall WB III, de Bakker PI, Word JM, Prisant MG, et al Structure validation by Calpha geometry: phi,psi and Cbeta deviation. Proteins. 2002; 50: 437–450. 10.1002/prot.10286 12557186

[29] Xu J, Zhang Y. How significant is a protein structure similarity with TM-score = 0.5? Bioinformatics. 2010; 26(7): 889–895 10.1093/bioinformatics/btq066 20164152 PMC2913670

[30] van Zundert GCP, Rodrigues JPGLM, Trellet M, Schmitz C, Kastritis PL, Karaca E. The HADDOCK2.2 webserver: User-friendly integrative modeling of biomolecular complexes. J. Mol. Biol. 2016; 428(4):720–725. 10.1016/j.jmb.2015.09.014 26410586

[31] BIOvIA, D. S. Discovery studio modeling environment. San Diego, Dassault Systemes, Release, 4; 2015. Available from: https://www.3dsbiovia.com/products/collaborative-science/biovia-discovery-studio/

[32] Xue LC, Rodrigues JP, Kastritis PL, Bonvin AM, Vangone A. PRODIGY: a webserver for predicting the binding affinity in protein-protein complexes. Bioinformatics. 2016; 32(23):3676–3678. 10.1093/bioinformatics/btw514 27503228

[33] Krissinel E, Henrick K. Inference of macromolecular assemblies from crystalline state. J. Mol. Biol. 2007; 372(3):774–97. 10.1016/j.jmb.2007.05.022 17681537

[34] Rodrigues CH, Pires DE, Ascher DB. DynaMut: Predicting the impact of mutations on protein conformation, flexibility and stability. Nucleic Acids Res. 2018; 46(W1):W350–W355. 10.1093/nar/gky300 29718330 PMC6031064

[35] Rogers AR, Harpending HC. Population growth makes waves in the distribution of pairwise genetic differences. Mol Biol Evol. 1992; 9 (3):552–569. 10.1093/oxfordjournals.molbev.a040727 1316531

[36] Yu WB, Tang GD, Zhang L, Corlett RT. Decoding the evolution and transmissions of the novel pneumonia coronavirus (SARS-CoV2/HCoV-19) using whole genomic data. Zool Res. 2020; 41(3):247–257. 10.24272/j.issn.2095-8137.2020.022 32351056 PMC7231477

[37] Dumaidi K, Al-Jawabreh A, Samarah F, Zraiqi A, Yaseen D. Genetic diversity of the enteroviruses detected from cerebrospinal fluid (CSF) samples of patients with suspected aseptic meningitis in northern West Bank, Palestine in 2017. PLoS ONE. 2018; 13(12): e0202243 10.1371/journal.pone.0202243 30532168 PMC6287809

[38] Brufsky A. Distinct Viral Clades of SARS-CoV-2: Implications for Modeling of Viral Spread. J Med Virol. 2020; 10.1002/jmv.25902 32311094 PMC7264516

[39] Banerjee AK, Begum F, Ray U. Mutation Hot Spots in Spike Protein of COVID-19. Preprints. 2020; 2020040281.

[40] Li W, Zhang C, Sui J, et al Receptor and viral determinants of SARS-coronavirus adaptation to human ACE2. EMBO J. 2005;24(8):1634‐1643. 10.1038/sj.emboj.7600640 15791205 PMC1142572

