# Supplementary material for: Time-lapse sentinel surveillance of SARS-CoV-2 spread in India
Source: PLoS One. 2020 Oct 22;15(10):e0241172. doi: 10.1371/journal.pone.0241172 (PMC7580942; doi:10.1371/journal.pone.0241172)
Supplement: S1 File — (DOC) [file pone.0241172.s001.doc]

**Manuscript #: Time-lapse sentinel surveillance of SARS-CoV-2 spread in India**

**Mukesh Thakur, Abhishek Singh, Bheem Dutt Joshi, Avijit Ghosh, Sujeet Kumar Singh, Neha Singh, Lalit Kumar Sharma and Kailash Chandra**

**E. mail: thamukesh@gmail.com**

This file includes: Table S1 to Table S10; Fig. S1 to Fig. S5.

Contents

[Table S1. Associated metadata for the SARS-CoV-2 genomes used in the present analysis 2](#__RefHeading___Toc52049861)

[Table S2. Summary of genetic diversity estimates and neutrality tests for demographic history of 112 SARS-CoV-2 genomes available from India. 5](#__RefHeading___Toc52049862)

[Table S3. Mutation frequency in three phases 5](#__RefHeading___Toc52049863)

[Table S4. Mutation frequency of each mutation 6](#__RefHeading___Toc52049864)

[Table S5. Mapping of mutations in the analyzed genomes. 7](#__RefHeading___Toc52049865)

[Table S6. Molecular docking analysis and binding affinities of mutant with ACE2 receptor 10](#__RefHeading___Toc52049866)

[Table S7. Ramachandran plot analysis of Mutant proteins 10](#__RefHeading___Toc52049867)

[Table S8. Quantitative assessment of similarity between mutant and Wild proteins 11](#__RefHeading___Toc52049868)

[Table S9. Interacting residues of RBD site with mutant spike protein 12](#__RefHeading___Toc52049869)

[Table S10. Mutation effect on stability and flexibility of protein 15](#__RefHeading___Toc52049870)

[Fig. S1. Molecular docking of the five selected mutants with human ACE2. 17](../../../../C:/Users/ZSI-CANNING/Supplementary%20materials_SARS-CoV-2%20spread_26092020_MT%20edits.doc#__RefHeading___Toc52049871)

[Fig. S2. Vibrational entropy change upon mutation affecting the flexibility of the protein. 18](#__RefHeading___Toc52049872)

[Fig. S3. Visual analysis of deformation energies of mutations S438F and R408I. 19](#__RefHeading___Toc52049873)

[Fig. S4. Visual analysis of deformation energies of mutations I434K and D614G. 20](#__RefHeading___Toc52049874)

[Fig. S5. Visual analysis of deformation energies of mutations K77M and A771V. 21](#__RefHeading___Toc52049875)

# Table S1. Associated metadata for the SARS-CoV-2 genomes used in the present analysis

| **S. No.** | **Accession ID** | **Haplotype_Set_1&2** | **Haplotypes_Set-3** | **Virus name** | **Location** | **Collection date** | **Additional location information** |
| --- | --- | --- | --- | --- | --- | --- | --- |
| 1 | EPI_ISL_420551 | 2 | 2 | hCoV-19/India/777/2020 | Asia / India | 2020-03-03 | Indian contact of Italian tourist |
| 2 | EPI_ISL_420550 | 2 | 2 | hCoV-19/India/2020773/2020 | Asia / India | 2020 | – |
| 3 | EPI_ISL_420553 | 2 | 2 | hCoV-19/India/781/2020 | Asia / India | 2020-03-03 | Italian tourist |
| 4 | EPI_ISL_420552 | 2 | 2 | hCoV-19/India/2020777/2020 | Asia / India | 2020 | – |
| 5 | EPI_ISL_420548 | 2 | 2 | hCoV-19/India/2020772/2020 | Asia / India | 2020 | – |
| 6 | EPI_ISL_420549 | 2 | 2 | hCoV-19/India/773/2020 | Asia / India | 2020-03-03 | Italian tourist |
| 7 | EPI_ISL_420544 | 2 | 2 | hCoV-19/India/2020763/2020 | Asia / India | 2020 | – |
| 8 | EPI_ISL_420546 | 2 | 2 | hCoV-19/India/2020770/2020 | Asia / India | 2020 | – |
| 9 | EPI_ISL_420554 | 2 | 2 | hCoV-19/India/2020781/2020 | Asia / India | 2020 | – |
| 10 | EPI_ISL_420545 | 3 | 3 | hCoV-19/India/770/2020 | Asia / India | 2020-03-03 | Italian tourist |
| 11 | EPI_ISL_420543 | 4 | 4 | hCoV-19/India/763/2020 | Asia / India | 2020-03-03 | Italian tourist |
| 12 | EPI_ISL_424365 | 5 | 5 | hCoV-19/India/3239/2020 | Asia / India | 2020-03-17 | Indian contact of Indian patient having travel history to Italy |
| 13 | EPI_ISL_424364 | 5 | 5 | hCoV-19/India/3118/2020 | Asia / India | 2020-03-17 | Indian contact of Indian patient having travel history to Italy |
| 14 | EPI_ISL_426414 | 6 | 6 | hCoV-19/India/GBRC1/2020 | Asia / India / Gujarat | 2020-04-05 | – |
| 15 | EPI_ISL_426415 | 7 | 7 | hCoV-19/India/GBRC1s/2020 | Asia / India / Gujarat | 2020-04-05 | – |
| 16 | EPI_ISL_426179 | 8 | 8 | hCoV-19/India/c31/2020 | Asia / India | 2020-03-02 | Indian contact of Indian patient having travel history to Italy |
| 17 | EPI_ISL_420555 | 8 | 8 | hCoV-19/India/c32/2020 | Asia / India | 2020-03-03 | Indian contact of Indian patient having travel history to Italy |
| 18 | EPI_ISL_420556 | 8 | 8 | hCoV-19/India/2020c32/2020 | Asia / India | 2020 | – |
| 19 | EPI_ISL_413522 | 9 | 9 | hCoV-19/India/1-27/2020 | Asia / India / Kerala | 2020-01-27 | Travel history to China |
| 20 | EPI_ISL_413523 | 10 | 10 | hCoV-19/India/1-31/2020 | Asia / India / Kerala | 2020-01-31 | Travel history to China |
| 21 | EPI_ISL_420547 | 11 | 11 | hCoV-19/India/772/2020 | Asia / India | 2020-03-03 | Italian tourist |
| 22 | EPI_ISL_424362 | 12 | 12 | hCoV-19/India/1135/2020 | Asia / India | 2020-03-10 | Indian citizen sampled at Iran |
| 23 | EPI_ISL_424363 | 13 | 13 | hCoV-19/India/1652/2020 | Asia / India | 2020-03-12 | Indian citizen sampled at Iran |
| 24 | EPI_ISL_424361 | 14 | 14 | hCoV-19/India/1063/2020 | Asia / India | 2020-03-10 | Indian citizen sampled at Iran |
| 25 | EPI_ISL_421664 | 15 | 15 | hCoV-19/India/1100/2020 | Asia / India | 2020-03-10 | Indian citizen sampled at Iran |
| 26 | EPI_ISL_421667 | 16 | 16 | hCoV-19/India/1115/2020 | Asia / India | 2020-03-10 | Indian citizen sampled at Iran |
| 27 | EPI_ISL_421672 | 17 | 17 | hCoV-19/India/1644/2020 | Asia / India | 2020-03-12 | Indian citizen sampled at Iran |
| 28 | EPI_ISL_421671 | 18 | 18 | hCoV-19/India/1621/2020 | Asia / India | 2020-03-12 | Indian citizen sampled at Iran |
| 29 | EPI_ISL_421669 | 19 | 19 | hCoV-19/India/1616/2020 | Asia / India | 2020-03-12 | Indian citizen sampled at Iran |
| 30 | EPI_ISL_421662 | 20 | 20 | hCoV-19/India/1073/2020 | Asia / India | 2020-03-10 | Indian citizen sampled at Iran |
| 31 | EPI_ISL_421663 | 21 | 21 | hCoV-19/India/1093/2020 | Asia / India | 2020-03-10 | Indian citizen sampled at Iran |
| 32 | EPI_ISL_421666 | 22 | 22 | hCoV-19/India/1111/2020 | Asia / India | 2020-03-10 | Indian citizen sampled at Iran |
| 33 | EPI_ISL_421665 | 23 | 23 | hCoV-19/India/1104/2020 | Asia / India | 2020-03-10 | Indian citizen sampled at Iran |
| 34 | EPI_ISL_421670 | 24 | 24 | hCoV-19/India/1617/2020 | Asia / India | 2020-03-12 | Indian citizen sampled at Iran |
| 35 | EPI_ISL_421668 | 25 | 25 | hCoV-19/India/1125/2020 | Asia / India | 2020-03-10 | Indian citizen sampled at Iran |
| 36 | EPI_ISL_417447 | 26 | 27 | hCoV-19/Italy/UniMI03/2020 | Europe / Italy / Lombardy / Milan | 2/24/2020 | 1/0/1900 |
| 37 | EPI_ISL_431102 | 27 | 27 | hCoV-19/India/GMC-KN318/2020 | Asia / India / Telangana / Hyderabad | 2020-03-11 | – |
| 38 | EPI_ISL_417445 | 27 | 26 | hCoV-19/Italy/UniMI01/2020 | Europe / Italy / Lombardy / Milan | 2/24/2020 | 1/0/1900 |
| 39 | EPI_ISL_435049 | 27 | 27 | hCoV-19/India/GBRC2/2020 | Asia / India / Gujarat / Ahmedabad | 2020-04-13 | – |
| 40 | EPI_ISL_435054 | 27 | 27 | hCoV-19/India/GBRC7/2020 | Asia / India / Gujarat / Ahmedabad | 2020-04-14 | – |
| 41 | EPI_ISL_435070 | 27 | 27 | hCoV-19/India/NCDC-01710/2020 | Asia / India / Delhi | 2020-03-18 | – |
| 42 | EPI_ISL_417446 | 28 | 28 | hCoV-19/Italy/UniMI02/2020 | Europe / Italy / Lombardy / Milan | 2/24/2020 | 1/0/1900 |
| 43 | EPI_ISL_424349 | 29 | 29 | hCoV-19/Iran/HGRC-01-IPI-8206/2020 | Asia / Iran | 3/9/2020 | 1/0/1900 |
| 44 | EPI_ISL_430466 | 30 | 30 | hCoV-19/India/S6/2020 | Asia / India / West Bengal / Tehatta | 2020-03-26 | – |
| 45 | EPI_ISL_431117 | 31 | 27 | hCoV-19/India/GMC-TC469/2020 | Asia / India / Telangana / Hyderabad | 2020-03-20 | – |
| 46 | EPI_ISL_430465 | 32 | 31 | hCoV-19/India/S5/2020 | Asia / India / West Bengal / Darjeeling | 2020-03-28 | – |
| 47 | EPI_ISL_430464 | 33 | 32 | hCoV-19/India/S3/2020 | Asia / India / West Bengal / Kolkata | 2020-03-21 | – |
| 48 | EPI_ISL_430468 | 33 | 32 | hCoV-19/India/S2/2020 | Asia / India / West_Bengal / Kolkata | 2020-03-21 | – |
| 49 | EPI_ISL_428479 | 34 | 33 | hCoV-19/India/nimh-0113/2020 | Asia / India / Karnataka | 2020-04-06 | – |
| 50 | EPI_ISL_430467 | 35 | 34 | hCoV-19/India/S11/2020 | Asia / India / West Bengal / East Medinipur | 2020-04-03 | – |
| 51 | EPI_ISL_428481 | 36 | 35 | hCoV-19/India/nimh-0130/2020 | Asia / India / Karnataka | 2020-04-06 | – |
| 52 | EPI_ISL_428482 | 37 | 36 | hCoV-19/India/nimh-0182/2020 | Asia / India / Karnataka | 2020-04-08 | – |
| 53 | EPI_ISL_428484 | 38 | 37 | hCoV-19/India/nimh-0351/2020 | Asia / India / Karnataka | 2020-04-10 | – |
| 54 | EPI_ISL_428487 | 39 | 38 | hCoV-19/India/nimh-1071/2020 | Asia / India / Karnataka | 2020-04-14 | – |
| 55 | EPI_ISL_428486 | 40 | 39 | hCoV-19/India/nimh-0996/2020 | Asia / India / Karnataka | 2020-04-14 | – |
| 56 | EPI_ISL_435074 |  | 40 | hCoV-19/India/NCDC-02250/2020 | Asia / India / West Bengal | 2020-03-28 | – |
| 57 | EPI_ISL_435075 |  | 40 | hCoV-19/India/NCDC-02331/2020 | Asia / India / Tamil Nadu | 2020-03-29 | – |
| 58 | EPI_ISL_435078 |  | 40 | hCoV-19/India/NCDC-02311/2020 | Asia / India / Tamil Nadu | 2020-03-29 | – |
| 59 | EPI_ISL_435080 |  | 40 | hCoV-19/India/NCDC-02334/2020 | Asia / India / Tamil Nadu | 2020-03-29 | – |
| 60 | EPI_ISL_435081 |  | 40 | hCoV-19/India/NCDC-02248/2020 | Asia / India / West Bengal | 2020-03-28 | – |
| 61 | EPI_ISL_435083 |  | 40 | hCoV-19/India/NCDC-02242/2020 | Asia / India / Tamil Nadu | 2020-03-28 | – |
| 62 | EPI_ISL_435084 |  | 40 | hCoV-19/India/NCDC-02309/2020 | Asia / India / Tamil Nadu | 2020-03-29 | – |
| 63 | EPI_ISL_435085 |  | 40 | hCoV-19/India/NCDC-02310/2020 | Asia / India / Mumbai | 2020-03-29 | – |
| 64 | EPI_ISL_435086 |  | 40 | hCoV-19/India/NCDC-02315/2020 | Asia / India / Mumbai | 2020-03-29 | – |
| 65 | EPI_ISL_435087 |  | 40 | hCoV-19/India/NCDC-02333/2020 | Asia / India / Tamil Nadu | 2020-03-29 | – |
| 66 | EPI_ISL_435090 |  | 40 | hCoV-19/India/NCDC-02326/2020 | Asia / India / Jammu | 2020-03-29 | – |
| 67 | EPI_ISL_435091 |  | 40 | hCoV-19/India/NCDC-02245/2020 | Asia / India / Tamil Nadu | 2020-03-28 | – |
| 68 | EPI_ISL_435092 |  | 40 | hCoV-19/India/NCDC-02244/2020 | Asia / India / Tamil Nadu | 2020-03-28 | – |
| 69 | EPI_ISL_435093 |  | 40 | hCoV-19/India/NCDC-02312/2020 | Asia / India / Tamil Nadu | 2020-03-29 | – |
| 70 | EPI_ISL_435094 |  | 40 | hCoV-19/India/NCDC-02327/2020 | Asia / India / Tamil Nadu | 2020-03-29 | – |
| 71 | EPI_ISL_435096 |  | 40 | hCoV-19/India/NCDC-02332/2020 | Asia / India / Tamil Nadu | 2020-03-29 | – |
| 72 | EPI_ISL_435097 |  | 40 | hCoV-19/India/NCDC-02252/2020 | Asia / India / West Bengal | 2020-03-28 | – |
| 73 | EPI_ISL_435098 |  | 40 | hCoV-19/India/NCDC-02240/2020 | Asia / India / Nepal | 2020-03-28 | – |
| 74 | EPI_ISL_435099 |  | 40 | hCoV-19/India/NCDC-02320/2020 | Asia / India / Uttar Pradesh | 2020-03-29 | – |
| 75 | EPI_ISL_435112 |  | 40 | hCoV-19/India/NCDC-02251/2020 | Asia / India / Bihar | 2020-03-28 | – |
| 76 | EPI_ISL_431103 | 41 | 40 | hCoV-19/India/GMC-KN443/2020 | Asia / India / Telangana / Hyderabad | 2020-03-16 | – |
| 77 | EPI_ISL_431101 | 42 | 41 | hCoV-19/India/GMC-RK100/2020 | Asia / India / Telangana / Hyderabad | 2020-03-01 | – |
| 78 | EPI_ISL_428483 | 43 | 42 | hCoV-19/India/nimh-0318/2020 | Asia / India / Karnataka | 2020-04-10 | – |
| 79 | EPI_ISL_435050 |  | 43 | hCoV-19/India/GBRC3/2020 | Asia / India / Gujarat / Ahmedabad | 2020-04-13 | – |
| 80 | EPI_ISL_435051 |  | 44 | hCoV-19/India/GBRC4/2020 | Asia / India / Gujarat / Ahmedabad | 2020-04-13 | – |
| 81 | EPI_ISL_435063 |  | 44 | hCoV-19/India/NCDC-01326/2020 | Asia / India / Delhi | 2020-03-13 | – |
| 82 | EPI_ISL_435064 |  | 44 | hCoV-19/India/NCDC-01705/2020 | Asia / India / Delhi | 2020-03-18 | – |
| 83 | EPI_ISL_435052 |  | 45 | hCoV-19/India/GBRC5/2020 | Asia / India / Gujarat / Ahmedabad | 2020-04-13 | – |
| 84 | EPI_ISL_435053 |  | 46 | hCoV-19/India/GBRC6/2020 | Asia / India / Gujarat / Ahmedabad | 2020-04-07 | – |
| 85 | EPI_ISL_435055 |  | 47 | hCoV-19/India/GBRC8/2020 | Asia / India / Gujarat / Gandhinagar | 2020-04-22 | – |
| 86 | EPI_ISL_435056 |  | 48 | hCoV-19/India/GBRC9/2020 | Asia / India / Gujarat / Mansa | 2020-04-21 | – |
| 87 | EPI_ISL_435062 |  | 49 | hCoV-19/India/NCDC-01538/2020 | Asia / India / Punjab | 2020-03-16 | – |
| 88 | EPI_ISL_435067 |  | 50 | hCoV-19/India/NCDC-01712/2020 | Asia / India / Delhi | 2020-03-18 | – |
| 89 | EPI_ISL_435065 |  | 51 | hCoV-19/India/NCDC-01475/2020 | Asia / India / Delhi | 2020-03-15 | – |
| 90 | EPI_ISL_435066 |  | 51 | hCoV-19/India/NCDC-01711/2020 | Asia / India / Delhi | 2020-03-18 | – |
| 91 | EPI_ISL_435068 |  | 51 | hCoV-19/India/NCDC-01757/2020 | Asia / India / Delhi | 2020-03-18 | – |
| 92 | EPI_ISL_435069 |  | 51 | hCoV-19/India/NCDC-01744/2020 | Asia / India / Delhi | 2020-03-18 | – |
| 93 | EPI_ISL_435061 |  | 52 | hCoV-19/India/NCDC-01537/2020 | Asia / India / Delhi | 2020-03-16 | – |
| 94 | EPI_ISL_435071 |  | 53 | hCoV-19/India/NCDC-02105/2020 | Asia / India / Delhi | 2020-03-25 | – |
| 95 | EPI_ISL_435072 |  | 54 | hCoV-19/India/NCDC-02155/2020 | Asia / India / Delhi | 2020-03-26 | – |
| 96 | EPI_ISL_435060 |  | 55 | hCoV-19/India/NCDC-01257/2020 | Asia / India / Uttar Pradesh / Noida | 2020-03-12 | – |
| 97 | EPI_ISL_435082 |  | 56 | hCoV-19/India/NCDC-02322/2020 | Asia / India / Uttar Pradesh | 2020-03-29 | – |
| 98 | EPI_ISL_435095 |  | 57 | hCoV-19/India/NCDC-02328/2020 | Asia / India / Tamil Nadu | 2020-03-29 | – |
| 99 | EPI_ISL_435100 |  | 58 | hCoV-19/India/NCDC-02321/2020 | Asia / India / Uttar Pradesh | 2020-03-29 | – |
| 100 | EPI_ISL_435088 |  | 59 | hCoV-19/India/NCDC-02336/2020 | Asia / India / Odisha | 2020-03-29 | – |
| 101 | EPI_ISL_435101 |  | 60 | hCoV-19/India/NCDC-01441/2020 | Asia / India / Ladakh | 2020-03-15 | – |
| 102 | EPI_ISL_435102 |  | 60 | hCoV-19/India/NCDC-01444/2020 | Asia / India / Ladakh | 2020-03-15 | – |
| 103 | EPI_ISL_435105 |  | 61 | hCoV-19/India/NCDC-01616/2020 | Asia / India / Ladakh | 2020-03-17 | – |
| 104 | EPI_ISL_435103 |  | 62 | hCoV-19/India/NCDC-01604/2020 | Asia / India / Ladakh | 2020-03-17 | – |
| 105 | EPI_ISL_435104 |  | 62 | hCoV-19/India/NCDC-01614/2020 | Asia / India / Ladakh | 2020-03-17 | – |
| 106 | EPI_ISL_435106 |  | 63 | hCoV-19/India/NCDC-01760/2020 | Asia / India / Ladakh | 2020-03-18 | – |
| 107 | EPI_ISL_435111 |  | 64 | hCoV-19/India/NCDC-02415/2020 | Asia / India / Delhi | 2020-03-30 | – |
| 108 | EPI_ISL_435108 |  | 65 | hCoV-19/India/NCDC-01457/2020 | Asia / India / Delhi | 2020-03-15 | – |
| 109 | EPI_ISL_435073 |  | 66 | hCoV-19/India/NCDC-02157/2020 | Asia / India / Delhi | 2020-03-26 | – |
| 110 | EPI_ISL_435079 |  | 67 | hCoV-19/India/NCDC-02318/2020 | Asia / India / Tamil Nadu | 2020-03-29 | – |
| 111 | EPI_ISL_435109 |  | 68 | hCoV-19/India/NCDC-01638/2020 | Asia / India / Delhi | 2020-03-17 | – |
| 112 | EPI_ISL_435110 |  | 69 | hCoV-19/India/NCDC-01501/2020 | Asia / India / Delhi | 2020-03-16 | – |
| 113 | EPI_ISL_435077 |  | 70 | hCoV-19/India/NCDC-02330/2020 | Asia / India / Maharashtra | 2020-03-29 | – |
| 114 | EPI_ISL_435076 |  | 71 | hCoV-19/India/NCDC-02329/2020 | Asia / India / Haryana | 2020-03-29 | – |
| 115 | EPI_ISL_435089 |  | 72 | hCoV-19/India/NCDC-02323/2020 | Asia / India / Andhra Pradesh | 2020-03-29 | – |
| 116 | EPI_ISL_435107 |  | 73 | hCoV-19/India/NCDC-02370/2020 | Asia / India / Kargil | 2020-03-29 | – |

# Table S2. Summary of genetic diversity estimates and neutrality tests for demographic history of 112 SARS-CoV-2 genomes available from India.

| Origin/ location | N | P | H | K | π | Hd | Tajima’s D | Fu’ Fs | SSD | Rg |
| --- | --- | --- | --- | --- | --- | --- | --- | --- | --- | --- |
| India | 112 | 143 | 72 | 6.9 | 0.00027 | 0.963 | -2.44849 * | -21.17755 ** | 0.00180 | 0.00425 |

Note: N, number of sequences; P, number of polymorphic sites; H, number of haplotypes; K, average nucleotide difference; π, nucleotide diversity; Hd, haplotype diversity; SSD, sums of squared deviations; Rg, Harpending’s raggedness index. * P < 0.01 (Tajima’s D); ** P < 0.00600 (Fu’s Fs); P = 0.9 (SSD); P = 0.87 (Rg).

# Table S3. Mutation frequency in three time frames

| **S.No** | **Phase** | **Mutation** | **Mutation Frequency** |
| --- | --- | --- | --- |
| 1 | T1 | R408I (22785G>T), A930V (24351C>T), D614G (23403A>G), D614G (23403A>G), D614G (23403A>G), I434K (22863T>A), D614G (23403A>G), D614G (23403A>G), D614G (23403A>G), D614G (23403A>G), D614G (23403A>G), T723I (23730C>T), D614G (23403A>G), G1124V (24933G>T), D614G (23403A>G), D614G (23403A>G), D614G (23403A>G), G1124V (24933G>T), K77M (21792A>T), A771V (23874C>T), V622I (23426G>A), Y28H (21644T>C) | 0.62 |
| 2 | T2 | R408I (22785G>T), A930V (24351C>T), D614G (23403A>G), D614G (23403A>G), D614G (23403A>G), I434K (22863T>A), D614G (23403A>G), D614G (23403A>G), D614G (23403A>G), D614G (23403A>G), D614G (23403A>G), T723I (23730C>T), D614G (23403A>G), G1124V (24933G>T), D614G (23403A>G)D614G (23403A>G), D614G (23403A>G), G1124V (24933G>T), K77M (21792A>T), A771V (23874C>T), V622I (23426G>A), Y28H (21644T>C), L5F (21575C>T) | 0.45 |
| 3 | T3 | R408I (22785G>T), A930V (24351C>T), V622I (23426G>A), D614G (23403A>G), D614G (23403A>G), D614G (23403A>G), D614G (23403A>G), D614G (23403A>G), D614G (23403A>G), D614G (23403A>G), D614G (23403A>G), D614G (23403A>G), D614G (23403A>G), P1263L (25350C>T), D614G (23403A>G), D614G (23403A>G), D614G (23403A>G), D614G (23403A>G), D614G (23403A>G), D614G (23403A>G), T299I (22458C>T), D614G (23403A>G), D614G (23403A>G), D614G (23403A>G), D614G (23403A>G), D614G (23403A>G), D614G (23403A>G), T1077S (24792C>G), D614G (23403A>G), D614G (23403A>G), D614G (23403A>G), D614G (23403A>G), D614G (23403A>G), D614G (23403A>G), D614G (23403A>G), D614G (23403A>G), D614G (23403A>G), D614G (23403A>G),  D614G (23403A>G), S438F (22875C>T),  D614G (23403A>G), S438F (22875C>T), D614G (23403A>G), I434K (22863T>A), D614G (23403A>G), D614G (23403A>G), D614G (23403A>G), D614G (23403A>G), Q271R (22374A>G), D614G (23403A>G), Q271R (22374A>G), D614G (23403A>G), D614G (23403A>G), G1124V (24933G>T), D614G (23403A>G), D614G (23403A>G), T723I (23730C>T), D614G (23403A>G), D614G (23403A>G), G1124V (24933G>T), D614G (23403A>G), D614G (23403A>G), D614G (23403A>G), D614G (23403A>G), D614G (23403A>G), C1250F (25311G>T), Y28H (21644T>C), K77M (21792A>T), A771V (23874C>T), L5F (21575C>T), L18F (21614C>T) | 0.64 |

# Table S4. Mutation frequency of each mutation

| **S.No** | **Phase** | **Mutation** | **Frequency** |
| --- | --- | --- | --- |
| 1 | **T1** | R408I (22785G>T) | 0.03 |
| 2 |  | A930V (24351C>T) | 0.03 |
| 3 |  | D614G (23403A>G) | 0.34 |
| 4 |  | I434K (22863T>A) | 0.03 |
| 5 |  | T723I (23730C>T) | 0.03 |
| 6 |  | G1124V (24933G>T) | 0.06 |
| 7 |  | K77M (21792A>T) | 0.03 |
| 8 |  | A771V (23874C>T) | 0.03 |
| 9 |  | V622I (23426G>A) | 0.03 |
| 10 |  | Y28H (21644T>C) | 0.02 |
| 1 | **T2** | R408I (22785G>T) | 0.02 |
| 2 |  | A930V (24351C>T) | 0.02 |
| 3 |  | D614G (23403A>G) | 0.34 |
| 4 |  | I434K (22863T>A) | 0.02 |
| 5 |  | T723I (23730C>T) | 0.02 |
| 6 |  | G1124V (24933G>T) | 0.06 |
| 7 |  | K77M (21792A>T) | 0.02 |
| 8 |  | A771V (23874C>T) | 0.02 |
| 9 |  | V622I (23426G>A) | 0.02 |
| 10 |  | Y28H (21644T>C) | 0.02 |
| 11 |  | L5F (21575C>T) | 0.02 |
| 1 | **T3** | R408I (22785G>T) | 0.01 |
| 2 |  | A930V (24351C>T) | 0.01 |
| 3 |  | V622I (23426G>A) | 0.01 |
| 4 |  | D614G (23403A>G) | 0.46 |
| 5 |  | P1263L (25350C>T) | 0.01 |
| 6 |  | T299I (22458C>T) | 0.01 |
| 7 |  | T1077S (24792C>G) | 0.01 |
| 8 |  | S438F (22875C>T) | 0.02 |
| 9 |  | I434K (22863T>A) | 0.01 |
| 10 |  | Q271R (22374A>G) | 0.02 |
| 11 |  | G1124V (24933G>T) | 0.02 |
| 12 |  | C1250F (25311G>T) | 0.01 |
| 13 |  | Y28H (21644T>C) | 0.01 |

# Table S5. Mapping of mutations in the analyzed genomes.

| **Accession ID** | **Travel History** | **Mutation** | **State** | **Hap** |
| --- | --- | --- | --- | --- |
| EPI_ISL_413522 | Wuhan | R408I (22785G>T) | Kerala | 9 |
| EPI_ISL_413523 | Wuhan | A930V (24351C>T) | Kerala | 10 |
| EPI_ISL_421671 | Iran | V622I (23426G>A) | Unknown | 18 |
| EPI_ISL_421669 | Iran | - | Unknown | 19 |
| EPI_ISL_421665 | Iran | - | Unknown | 23 |
| EPI_ISL_421672 | Iran | - | Unknown | 17 |
| EPI_ISL_424363 | Iran | - | Unknown | 13 |
| EPI_ISL_421670 | Iran | - | Unknown | 24 |
| EPI_ISL_421663 | Iran | - | Unknown | 21 |
| EPI_ISL_421667 | Iran | - | Unknown | 16 |
| EPI_ISL_421664 | Iran | - | Unknown | 15 |
| EPI_ISL_421662 | Iran | - | Unknown | 20 |
| EPI_ISL_424361 | Iran | - | Unknown | 14 |
| EPI_ISL_421666 | Iran | - | Unknown | 22 |
| EPI_ISL_421668 | Iran | - | Unknown | 25 |
| EPI_ISL_428483 | Unknown | - | Karnataka | 42 |
| EPI_ISL_428484 | Unknown | - | Karnataka | 37 |
| EPI_ISL_428486 | Unknown | - | Karnataka | 39 |
| EPI_ISL_428487 | Unknown | - | Karnataka | 38 |
| EPI_ISL_435074 | Unknown | - | West Bengal | 40 |
| EPI_ISL_435079 | Unknown | - | Tamil Nadu | 67 |
| EPI_ISL_435081 | Unknown | - | West Bengal | 40 |
| EPI_ISL_435082 | Unknown | - | Uttar Pradesh | 56 |
| EPI_ISL_435083 | Unknown | - | Tamil Nadu | 40 |
| EPI_ISL_435085 | Unknown | - | Mumbai | 40 |
| EPI_ISL_435086 | Unknown | - | Mumbai | 40 |
| EPI_ISL_435089 | Unknown | - | Andhra Pradesh | 72 |
| EPI_ISL_435090 | Unknown | - | Jammu | 40 |
| EPI_ISL_435091 | Unknown | - | Tamil Nadu | 40 |
| EPI_ISL_435095 | Unknown | - | Tamil Nadu | 57 |
| EPI_ISL_435097 | Unknown | - | West Bengal | 40 |
| EPI_ISL_435099 | Unknown | - | Uttar Pradesh | 40 |
| EPI_ISL_435100 | Unknown | - | Uttar Pradesh | 58 |
| EPI_ISL_435101 | Unknown | - | Ladakh | 60 |
| EPI_ISL_435105 | Unknown | - | Ladakh | 61 |
| EPI_ISL_435049 | Unknown | D614G (23403A>G) | Gujarat | 27 |
| EPI_ISL_435050 | Unknown | D614G (23403A>G) | Gujarat | 43 |
| EPI_ISL_435051 | Unknown | D614G (23403A>G) | Gujarat | 44 |
| EPI_ISL_435052 | Unknown | D614G (23403A>G) | Gujarat | 45 |
| EPI_ISL_435053 | Unknown | D614G (23403A>G) | Gujarat | 46 |
| EPI_ISL_435054 | Unknown | D614G (23403A>G) | Gujarat | 27 |
| EPI_ISL_435055 | Unknown | D614G (23403A>G) | Gujarat | 47 |
| EPI_ISL_435056 | Unknown | D614G (23403A>G) | Gujarat | 48 |
| EPI_ISL_435060 | Unknown | D614G (23403A>G), | Uttar Pradesh | 55 |
| EPI_ISL_435061 | Unknown | D614G (23403A>G), P1263L (25350C>T) | Delhi | 52 |
| EPI_ISL_435062 | Unknown | D614G (23403A>G) | Punjab | 49 |
| EPI_ISL_435063 | Unknown | D614G (23403A>G) | Delhi | 44 |
| EPI_ISL_435064 | Unknown | D614G (23403A>G) | Delhi | 44 |
| EPI_ISL_435065 | Unknown | D614G (23403A>G) | Delhi | 51 |
| EPI_ISL_435066 | Unknown | D614G (23403A>G), | Delhi | 51 |
| EPI_ISL_435067 | Unknown | D614G (23403A>G), T299I (22458C>T) | Delhi | 50 |
| EPI_ISL_435068 | Unknown | D614G (23403A>G) | Delhi | 51 |
| EPI_ISL_435069 | Unknown | D614G (23403A>G) | Delhi | 51 |
| EPI_ISL_435070 | Unknown | D614G (23403A>G) | Delhi | 27 |
| EPI_ISL_435071 | Unknown | D614G (23403A>G) | Delhi | 53 |
| EPI_ISL_435072 | Unknown | D614G (23403A>G) | Delhi | 54 |
| EPI_ISL_435073 | Unknown | D614G (23403A>G), T1077S (24792C>G) | Delhi | 66 |
| EPI_ISL_420545 | Italian Tourist | D614G (23403A>G) | Unknown | 3 |
| EPI_ISL_420544 | Unknown | D614G (23403A>G) | Unknown | 2 |
| EPI_ISL_420546 | Unknown | D614G (23403A>G) | Unknown | 2 |
| EPI_ISL_420552 | Unknown | D614G (23403A>G) | Unknown | 2 |
| EPI_ISL_420553 | Italian Tourist | D614G (23403A>G) | Unknown | 2 |
| EPI_ISL_420548 | Unknown | D614G (23403A>G) | Unknown | 2 |
| EPI_ISL_420551 | Contact with Italian Tourist | D614G (23403A>G) | Unknown | 2 |
| EPI_ISL_420549 | Italian Tourist | D614G (23403A>G) | Unknown | 2 |
| EPI_ISL_420550 | Unknown | D614G (23403A>G) | Unknown | 2 |
| EPI_ISL_420554 | Unknown | D614G (23403A>G) | Unknown | 2 |
| EPI_ISL_420543 | Italian Tourist | D614G (23403A>G), S438F (22875C>T) | Unknown | 4 |
| EPI_ISL_420547 | Italian Tourist | D614G (23403A>G), S438F (22875C>T) | Unknown | 11 |
| EPI_ISL_424362 | Iran | D614G (23403A>G), I434K (22863T>A) | Unknown | 12 |
| EPI_ISL_420555 | Italy | D614G (23403A>G) | Unknown | 8 |
| EPI_ISL_420556 | Unknown | D614G (23403A>G) | Unknown | 8 |
| EPI_ISL_424365 | Italy | D614G (23403A>G) | Unknown | 5 |
| EPI_ISL_424364 | Contact with Italian Tourist | D614G (23403A>G) | Unknown | 5 |
| EPI_ISL_426414 | Spain | D614G (23403A>G), Q271R (22374A>G) | Gujarat | 6 |
| EPI_ISL_426415 | Spain | D614G (23403A>G), Q271R (22374A>G) | Gujarat | 7 |
| EPI_ISL_426179 | Contact with Italian Tourist | D614G (23403A>G) | Unknown | 8 |
| EPI_ISL_430464 | While traveling | D614G (23403A>G), G1124V (24933G>T) | West Bengal | 32 |
| EPI_ISL_430465 | While traveling | D614G (23403A>G) | West Bengal | 31 |
| EPI_ISL_430466 | While traveling | D614G (23403A>G), T723I (23730C>T) | West Bengal | 30 |
| EPI_ISL_430467 | While traveling | D614G (23403A>G) | West Bengal | 34 |
| EPI_ISL_430468 | While traveling | D614G (23403A>G), G1124V (24933G>T) | West Bengal | 32 |
| EPI_ISL_431102 | Visit to italy | D614G (23403A>G) | Telangana | 27 |
| EPI_ISL_431117 | Visit to UK | D614G (23403A>G) | Telangana | 27 |
| EPI_ISL_428479 | Unknown | D614G (23403A>G) | Karnataka | 33 |
| EPI_ISL_428481 | Unknown | D614G (23403A>G) | West Bengal | 35 |
| EPI_ISL_428482 | Unknown | D614G (23403A>G), C1250F (25311G>T) | Karnataka | 36 |
| EPI_ISL_431101 | Dubai Visit | Y28H (21644T>C) | Telangana | 41 |
| EPI_ISL_435075 | Unknown | K77M (21792A>T), A771V (23874C>T) | Tamil Nadu | 40 |
| EPI_ISL_435076 | Unknown | - | Haryana | 71 |
| EPI_ISL_435077 | Unknown | - | Maharashtra | 70 |
| EPI_ISL_435078 | Unknown | - | Tamil Nadu | 40 |
| EPI_ISL_435080 | Unknown | - | Tamil Nadu | 40 |
| EPI_ISL_435084 | Unknown | - | Tamil Nadu | 40 |
| EPI_ISL_435087 | Unknown | - | Tamil Nadu | 40 |
| EPI_ISL_435088 | Unknown | - | Odisha | 59 |
| EPI_ISL_435111 | Unknown | - | Delhi | 64 |
| EPI_ISL_435092 | Unknown | - | Tamil Nadu | 40 |
| EPI_ISL_435093 | Unknown |  | Tamil Nadu | 40 |
| EPI_ISL_435094 | Unknown | - | Tamil Nadu | 40 |
| EPI_ISL_435096 | Unknown | - | Tamil Nadu | 40 |
| EPI_ISL_435098 | Unknown | - | Nepal | 40 |
| EPI_ISL_435104 | Unknown | - | Ladakh | 62 |
| EPI_ISL_435106 | Unknown | - | Ladakh | 63 |
| EPI_ISL_435108 | Unknown | L5F (21575C>T) | Delhi | 65 |
| EPI_ISL_435109 | Unknown | - | Delhi | 68 |
| EPI_ISL_435110 | Unknown | L18F (21614C>T) | Delhi | 69 |
| EPI_ISL_435112 | Unknown | - | Bihar | 40 |
| EPI_ISL_435102 | Unknown | - | Ladakh | 60 |
| EPI_ISL_435103 | Unknown | - | Ladakh | 62 |
| EPI_ISL_435107 | Unknown | - | Kargil | 73 |

# Table S6. Molecular docking analysis and binding affinities of mutant with ACE2 receptor

| **Parameters** | **Wild** | **M1** | **M2** | **M3** | **M4** | **M5** |
| --- | --- | --- | --- | --- | --- | --- |
| HADDOCK score | 106.6 +/- 15.7 | 116.9 +/- 9.3 | 26.5 +/- 15.7 | 118.4 +/- 13.2 | 131.7 +/- 18.6 | 127.7 +/- 20.1 |
| Z-Score | -1.3 | -1.5 | -2 | -2.2 | -1.5 | -1.4 |
| Binding Affinity (ΔG) kcal mol-1 | -13.2 | -12.7 | -12.7 | -13 | -15.4 | -14.2 |
| Dissociation constant (Kd) | 2.2E-10 | 4.8E-10 | 4.5E-10 | 3.1E-10 | 5.3E-12 | 4.1E-11 |

# Table S7. Ramachandran plot analysis of Mutant proteins

| **Rampage** | **Wild** | **M1** | **M2** | **M3** | **M4** | **M5** |
| --- | --- | --- | --- | --- | --- | --- |
| Favored region | 96.30% | 95.30% | 93.90% | 96.50% | 96.50% | 95.60% |
| Allowed region | 2.60% | 3.50% | 4.60% | 2.70% | 2.70% | 3.60% |
| Outlier region | 1.10% | 1.20% | 1.50% | 0.80% | 0.80% | 0.80% |

# Table S8. Quantitative assessment of similarity between mutant and Wild proteins

| **Mutants/Wild** | **RMSD** | **Tmscore** |
| --- | --- | --- |
| M1 | 1.966 | 0.9665 |
| M2 | 1.577 | 0.9869 |
| M3 | 1.223 | 0.9788 |
| M4 | 1.22 | 0.9789 |
| M5 | 0.812 | 0.9823 |

# Table S9. Interacting residues of RBD site with mutant spike protein

|  | **S. No.** | **Hydrogen Bond** | | | **Salt Bridge** | | |
| --- | --- | --- | --- | --- | --- | --- | --- |
|  |  | [**Structure 1**](javascript:openWindow('pi_ipage_atom1.html',400,250);) | [**Dist. [Å]**](javascript:openWindow('pi_ipage_atmdist.html',400,250);) | [**Structure 2**](javascript:openWindow('pi_ipage_atom2.html',400,250);) | [**Structure 1**](javascript:openWindow('pi_ipage_atom1.html',400,250);) | [**Dist. [Å]**](javascript:openWindow('pi_ipage_atmdist.html',400,250);) | [**Structure 2**](javascript:openWindow('pi_ipage_atom2.html',400,250);) |
| Wild | 1 | A:TYR  50[ HH ] | 1.93 | B:GLU 465[ OE2] | A:GLU  56[ OE1] | 2.71 | B:LYS 424[ NZ ] |
|  | 2 | A:GLN  60[HE22] | 2.08 | B:PHE 515[ O  ] | A:GLU  56[ OE2] | 2.77 | B:LYS 424[ NZ ] |
|  | 3 | A:ASN  64[HD22] | 1.62 | B:GLU 516[ OE2] | A:ASP  67[ OD1] | 3.96 | B:ARG 357[ NE ] |
|  | 4 | A:LYS  74[ HZ3] | 1.70 | B:ASN 360[ OD1] | A:ASP  67[ OD1] | 2.61 | B:ARG 357[ NH2] |
|  | 5 | A:LYS 114[ HZ3] | 1.87 | B:ASN 334[ OD1] |  | | |
|  | 6 | A:LYS 114[ HZ1] | 1.72 | B:LEU 335[ O  ] |  |  |  |
|  | 7 | A:THR 125[ OG1] | 3.26 | B:ARG 355[ O  ] |  |  |  |
|  | 8 | A:LYS 131[ HZ1] | 1.84 | B:SER 349[ OG ] |  |  |  |
|  | 9 | A:GLN 139[HE21] | 1.79 | B:SER 494[ OG ] |  |  |  |
|  | 10 | A:GLU  56[ OE1] | 3.32 | B:ASP 427[ N  ] |  |  |  |
|  | 11 | A:ASN  63[ OD1] | 2.02 | B:TYR 396[ HH ] |  |  |  |
|  | 12 | A:ASP  67[ OD1] | 1.63 | B:ARG 357[HH22] |  |  |  |
|  | 13 | A:GLU 110[ OE2] | 2.89 | B:ASN 334[ N  ] |  |  |  |
|  | 14 | A:ASN 121[ OD1] | 2.77 | B:ARG 357[ N  ] |  |  |  |
|  | 15 | A:THR 125[ OG1] | 3.90 | B:ARG 355[ N  ] |  |  |  |
|  | 16 | A:SER 128[ OG ] | 1.79 | B:ARG 466[HH22] |  |  |  |
|  | 17 | A:ASN 338[ O  ] | 1.70 | B:LYS 462[ HZ3] |  |  |  |
|  | 18 | A:GLN 340[ O  ] | 2.03 | B:LYS 462[ HZ1] |  |  |  |
| M1 | 1 | A:ARG 559[ HE ] | 1.72 | B:GLU 340[ OE1] | A:ARG 559[ NE ] | 2.61 | B:GLU 340[ OE1] |
|  | 2 | A:GLN 388[HE21] | 2.36 | B:GLU 340[ OE2] | A:ARG 559[ NH2] | 3.16 | B:GLU 340[ OE1] |
|  | 3 | A:ARG 559[HH21] | 1.58 | B:GLU 340[ OE2] | A:ARG 559[ NE ] | 3.48 | B:GLU 340[ OE2] |
|  | 4 | A:LEU 320[ N  ] | 3.39 | B:ASN 360[ OD1] | A:ARG 559[ NH2] | 2.63 | B:GLU 340[ OE2] |
|  | 5 | A:GLN  42[HE22] | 2.29 | B:PRO 463[ O  ] | A:ASP  30[ OD2] | 3.79 | B:ARG 346[ NE ] |
|  | 6 | A:LYS  31[ HZ1] | 2.35 | B:PRO 491[ O  ] | A:ASP  30[ OD1] | 2.92 | B:ARG 346[ NH1] |
|  | 7 | A:GLN 552[ OE1] | 2.27 | B:ASN 334[HD21] | A:ASP  30[ OD2] | 2.68 | B:ARG 346[ NH1] |
|  | 8 | A:GLN 552[ OE1] | 3.87 | B:LEU 335[ N  ] | A:ASP  38[ OD2] | 2.68 | B:ARG 466[ NH1] |
|  | 9 | A:ASP  30[ OD2] | 1.66 | B:ARG 346[HH12] | A:ASP  38[ OD2] | 3.17 | B:ARG 466[ NH2] |
|  | 10 | A:ALA 387[ O  ] | 1.68 | B:LYS 356[ HZ2] |  | | |
|  | 11 | A:MET 383[ SD ] | 2.35 | B:ARG 357[HH22] |  |  |  |
|  | 12 | A:ASN 322[ O  ] | 1.65 | B:ARG 357[HH21] |  |  |  |
|  | 13 | A:THR  27[ OG1] | 2.13 | B:LYS 444[ HZ1] |  |  |  |
|  | 14 | A:GLU  23[ OE2] | 2.67 | B:VAL 445[ N  ] |  |  |  |
|  | 15 | A:ASP  38[ OD2] | 1.84 | B:ARG 466[HH11] |  |  |  |
| M2 | 1 | A:LYS  74[ HZ3] | 2.09 | B:GLU 465[ OE1] | A:LYS  74[ NZ ] | 2.62 | B:GLU 465[ OE1] |
|  | 2 | A:LYS  74[ HZ2] | 1.73 | B:GLU 465[ OE2] | A:LYS  74[ NZ ] | 2.71 | B:GLU 465[ OE2] |
|  | 3 | A:LYS  74[ HZ1] | 1.66 | B:PRO 463[ O  ] | A:ARG 115[ NH1] | 3.75 | B:ASP 428[ OD1] |
|  | 4 | A:LYS 114[ HZ3] | 2.24 | B:SER 514[ OG ] | A:ARG 115[ NH1] | 3.16 | B:ASP 428[ OD2] |
|  | 5 | A:ARG 115[HH11] | 2.26 | B:ASP 428[ OD2] | A:ARG 115[ NH2] | 2.58 | B:ASP 428[ OD1] |
|  | 6 | A:ARG 115[HH22] | 1.56 | B:ASP 428[ OD1] | A:ARG 115[ NH2] | 3.33 | B:ASP 428[ OD2] |
|  | 7 | A:ASN 137[HD22] | 2.18 | B:PHE 562[ O  ] | A:ASP  67[ OD1] | 2.66 | B:ARG 355[ NH2] |
|  | 8 | A:GLU 140[ N  ] | 3.85 | B:PHE 562[ O  ] | A:ASP  67[ OD1] | 2.72 | B:ARG 355[ NE ] |
|  | 9 | A:ASP  67[ OD1] | 1.74 | B:ARG 355[HH21] | A:GLU 110[ OE1] | 3.72 | B:ARG 457[ NH2] |
|  | 10 | A:ASP  67[ OD1] | 1.86 | B:ARG 355[ HE ] | A:GLU 110[ OE2] | 3.07 | B:ARG 457[ NH2] |
|  | 11 | A:ASP  67[ OD2] | 2.60 | B:ARG 355[ N  ] | A:GLU 110[ OE2] | 2.66 | B:ARG 457[ NH1] |
|  | 12 | A:GLN  81[ OE1] | 2.32 | B:LYS 462[ HZ2] |  | | |
|  | 13 | A:GLU 110[ OE2] | 1.69 | B:ARG 457[HH12] |  |  |  |
|  | 14 | A:ASN 121[ OD1] | 1.69 | B:ARG 357[HH22] |  |  |  |
|  | 15 | A:GLN 139[ OE1] | 3.24 | B:PHE 565[ N  ] |  |  |  |
| M3 | 1 | A:ARG 559[HH12] | 1.77 | B:ALA 352[ O  ] | A:LYS  26[ NZ ] | 3.87 | B:ASP 428[ OD1] |
|  | 2 | A:ARG 559[HH22] | 1.55 | B:TRP 353[ O  ] | A:LYS  26[ NZ ] | 2.64 | B:ASP 428[ OD2] |
|  | 3 | A:LYS  26[ HZ1] | 1.60 | B:ASP 428[ OD2] | A:LYS  26[ NZ ] | 3.98 | B:GLU 516[ OE1] |
|  | 4 | A:THR  92[ N  ] | 2.69 | B:GLU 465[ OE2] | A:LYS  26[ NZ ] | 2.62 | B:GLU 516[ OE2] |
|  | 5 | A:LYS  26[ HZ3] | 1.66 | B:GLU 516[ OE2] | A:GLU  37[ OE2] | 3.91 | B:ARG 357[ NE ] |
|  | 6 | A:GLN 552[ OE1] | 2.11 | B:ARG 346[HH12] | A:GLU  37[ OE2] | 3.18 | B:ARG 357[ NH1] |
|  | 7 | A:THR 548[ O  ] | 1.95 | B:ARG 346[HH11] | A:GLU  37[ OE2] | 3.15 | B:ARG 357[ NH2] |
|  | 8 | A:TYR 215[ OH ] | 2.22 | B:TYR 351[ HH ] | A:ASP 213[ OD2] | 2.55 | B:LYS 458[ NZ ] |
|  | 9 | A:ALA 387[ O  ] | 2.75 | B:ARG 357[ N  ] | A:ASP 213[ OD1] | 2.57 | B:LYS 462[ NZ ] |
|  | 10 | A:ASN  33[ O  ] | 2.30 | B:ARG 357[HH12] | A:GLU 564[ OE2] | 2.60 | B:ARG 466[ NE ] |
|  | 11 | A:ASN  33[ O  ] | 1.89 | B:ARG 357[HH21] | A:GLU 564[ OE2] | 2.87 | B:ARG 466[ NH2] |
|  | 12 | A:ASP  30[ OD1] | 2.82 | B:ASN 394[ N  ] |  | | |
|  | 13 | A:ASP  30[ OD2] | 1.68 | B:TYR 396[ HH ] |  |  |  |
|  | 14 | A:ASP 213[ OD2] | 1.52 | B:LYS 458[ HZ3] |  |  |  |
|  | 15 | A:GLY 211[ O  ] | 1.75 | B:LYS 462[ HZ1] |  |  |  |
|  | 16 | A:ASP 213[ OD1] | 1.52 | B:LYS 462[ HZ2] |  |  |  |
|  | 17 | A:GLU 564[ OE2] | 1.67 | B:ARG 466[ HE ] |  |  |  |
|  | 18 | A:SER 563[ OG ] | 1.64 | B:ARG 466[HH21] |  |  |  |
|  | 19 | A:SER 563[ O  ] | 2.33 | B:ARG 466[HH22] |  |  |  |
|  | 20 | A:GLU 564[ OE2] | 2.04 | B:ARG 466[HH22] |  |  |  |
| M4 | 1 | A:LYS 353[ HZ2] | 2.17 | B:TYR 351[ O  ] | A:ASP  30[ OD1] | 3.79 | B:ARG 346[ NE ] |
|  | 2 | A:GLN 552[HE21] | 2.15 | B:ASN 360[ OD1] | A:ASP  30[ OD1] | 2.72 | B:ARG 346[ NH1] |
|  | 3 | A:GLN 325[HE22] | 2.31 | B:ASP 428[ OD2] | A:GLU  37[ OE1] | 3.84 | B:ARG 466[ NH2] |
|  | 4 | A:GLN 325[ N  ] | 3.35 | B:GLU 465[ OE1] | A:GLU  37[ OE2] | 2.75 | B:ARG 466[ NH2] |
|  | 5 | A:GLY 326[ N  ] | 2.70 | B:GLU 465[ OE1] |  | | |
|  | 6 | A:ASP  30[ OD1] | 1.71 | B:ARG 346[HH11] |  |  |  |
|  | 7 | A:THR  27[ OG1] | 1.78 | B:ARG 346[HH22] |  |  |  |
|  | 8 | A:ASN 556[ OD1] | 2.26 | B:ASN 360[HD21] |  |  |  |
|  | 9 | A:ASP  30[ OD2] | 2.39 | B:ASN 450[HD22] |  |  |  |
|  | 10 | A:ASN 330[ OD1] | 1.68 | B:LYS 462[ HZ3] |  |  |  |
|  | 11 | A:GLU 329[ O  ] | 1.92 | B:LYS 462[ HZ2] |  |  |  |
|  | 12 | A:GLU  37[ OE2] | 2.18 | B:ARG 466[HH21] |  |  |  |
| M5 | 1 | A:LYS  74[ HZ2] | 1.96 | B:ARG 355[ O  ] | A:LYS 114[ NZ ] | 2.59 | B:GLU 516[ OE1] |
|  | 2 | A:LYS  74[ HZ2] | 2.47 | B:LYS 356[ O  ] | A:ARG 115[ NH1] | 3.04 | B:ASP 428[ OD1] |
|  | 3 | A:THR  78[ OG1] | 3.89 | B:TRP 353[ O  ] | A:ARG 115[ NH2] | 3.94 | B:ASP 428[ OD1] |
|  | 4 | A:GLU 110[ N  ] | 2.86 | B:TYR 423[ OH ] | A:GLU  75[ OE1] | 3.47 | B:LYS 356[ NZ ] |
|  | 5 | A:LYS 114[ HZ3] | 1.62 | B:GLU 516[ OE1] | A:GLU  75[ OE2] | 2.62 | B:LYS 356[ NZ ] |
|  | 6 | A:LYS 114[ HZ2] | 2.23 | B:PHE 515[ O  ] |  | | |
|  | 7 | A:LYS 114[ HZ2] | 2.20 | B:THR 430[ O  ] |  |  |  |
|  | 8 | A:ARG 115[HH11] | 1.99 | B:ASP 428[ OD1] |  |  |  |
|  | 9 | A:GLU  75[ OE2] | 1.58 | B:LYS 356[ HZ3] |  |  |  |
|  | 10 | A:GLU  75[ O  ] | 2.39 | B:ASN 354[HD22] |  |  |  |
|  | 11 | A:THR  78[ OG1] | 1.73 | B:ARG 466[HH12] |  |  |  |
|  | 12 | A:THR  78[ OG1] | 3.47 | B:ARG 355[ N  ] |  |  |  |
|  | 13 | A:ASN 103[ OD1] | 2.30 | B:ARG 466[ HE ] |  |  |  |
|  | 14 | A:ASN 103[ OD1] | 1.77 | B:ARG 466[HH21] |  |  |  |
|  | 15 | A:SER 106[ O  ] | 1.81 | B:ARG 355[ HE ] |  |  |  |
|  | 16 | A:VAL 107[ O  ] | 1.78 | B:ARG 355[HH21] |  |  |  |
|  | 17 | A:VAL 107[ O  ] | 3.44 | B:ARG 466[ N  ] |  |  |  |
|  | 18 | A:GLU 110[ OE2] | 3.28 | B:SER 514[ N  ] |  |  |  |
|  | 19 | A:SER 113[ O  ] | 1.87 | B:TYR 396[ HH ] |  |  |  |

# Table S10. Mutation effect on stability and flexibility of protein

| **Mutation** | **ΔΔG kcal/mol** | **Stability** | **ΔΔSVib ENCoM kcal.mol-1.K-1** | **Flexibility** |
| --- | --- | --- | --- | --- |
| S438F | 0.92 | Stabilizing | -0.365 | Decrease |
| R408I | 0.527 | Stabilizing | 0.047 | Increase |
| I434K | -1.699 | Destabilizing | 0.045 | Increase |
| D614G | 0.776 | Stabilizing | 0.008 | Increase |
| K77M | 0.396 | Stabilizing | -0.506 | Decrease |
| A771V | 0.491 | Stabilizing | -0.361 | Decrease |

| 14 |  | K77M (21792A>T) | 0.01 |
| --- | --- | --- | --- |
| 15 |  | A771V (23874C>T) | 0.01 |
| 16 |  | L5F (21575C>T) | 0.01 |
| 17 |  | L18F (21614C>T) | 0.01 |

**
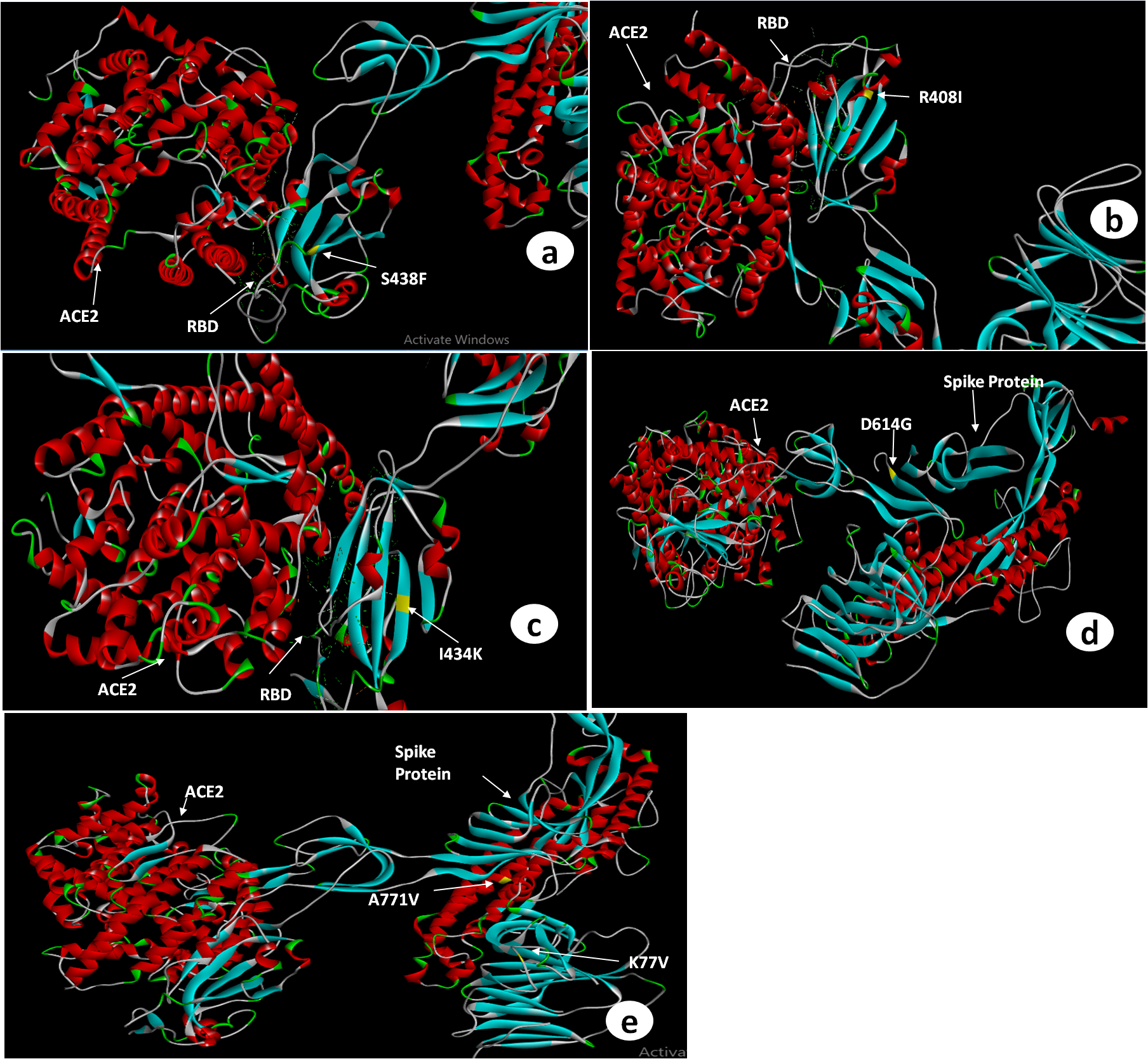
**

Fig. S1. Molecular docking of the five selected mutants with human ACE2. (A) M1-S438F+D614G, (B) M2-R408I and (C) M3-I434K+D614G carrying mutations in the RBD region (RBD: 336-516). (D) M4-D614G was most prevalent and carrying a mutation in the S1 domain of Spike protein (S1:515-667) and (E) M5K77M+A771V carrying mutations outside both RBD and S1 domains but occurred in a widely spread haplotype H-40 observed in 11 States of India.


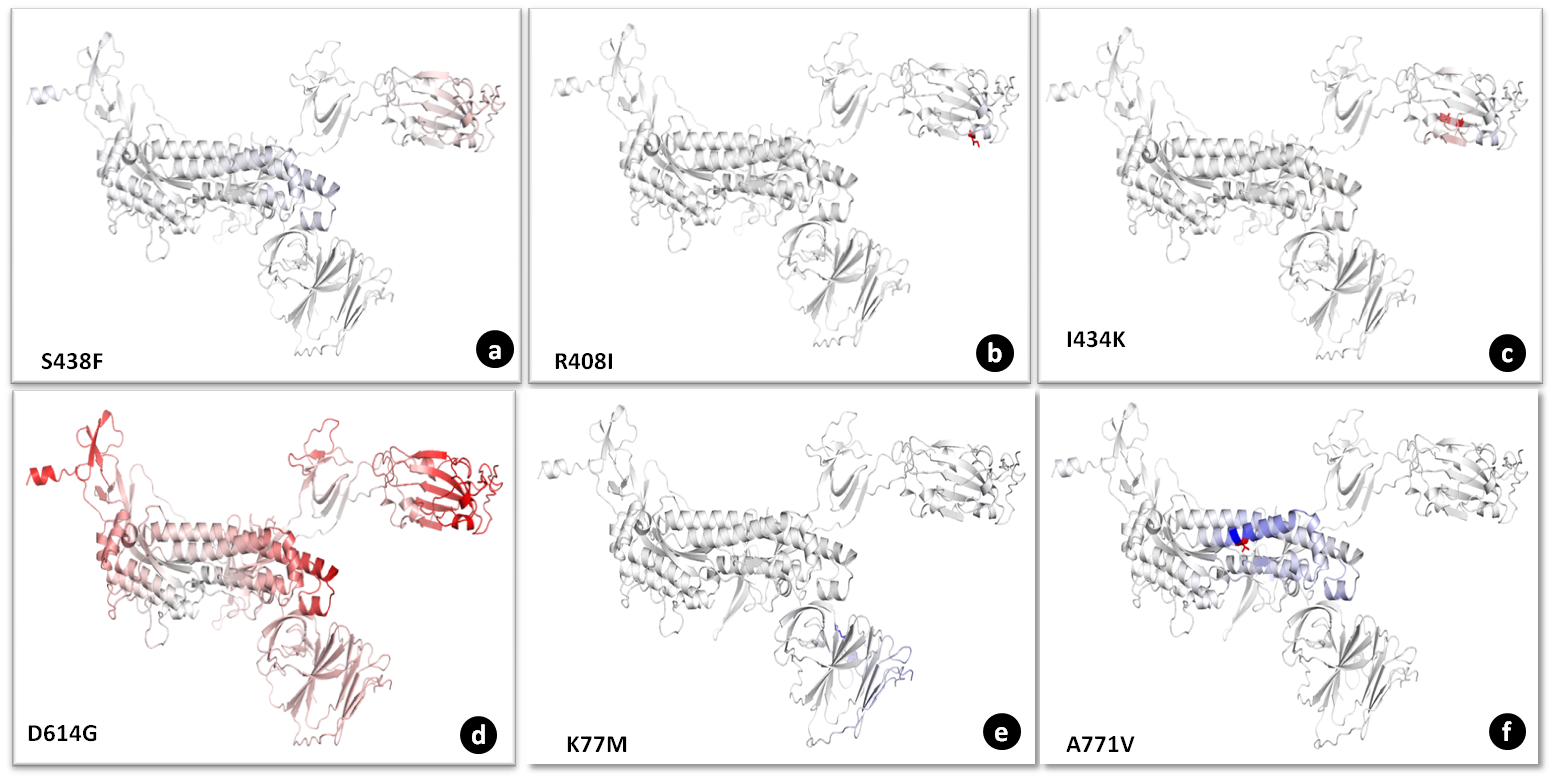


##### ***Fig. S2. Vibrational entropy change upon mutation affecting the flexibility of the protein.* BLUE color represented rigidification of the structure and RED color denoted a gain in flexibility**


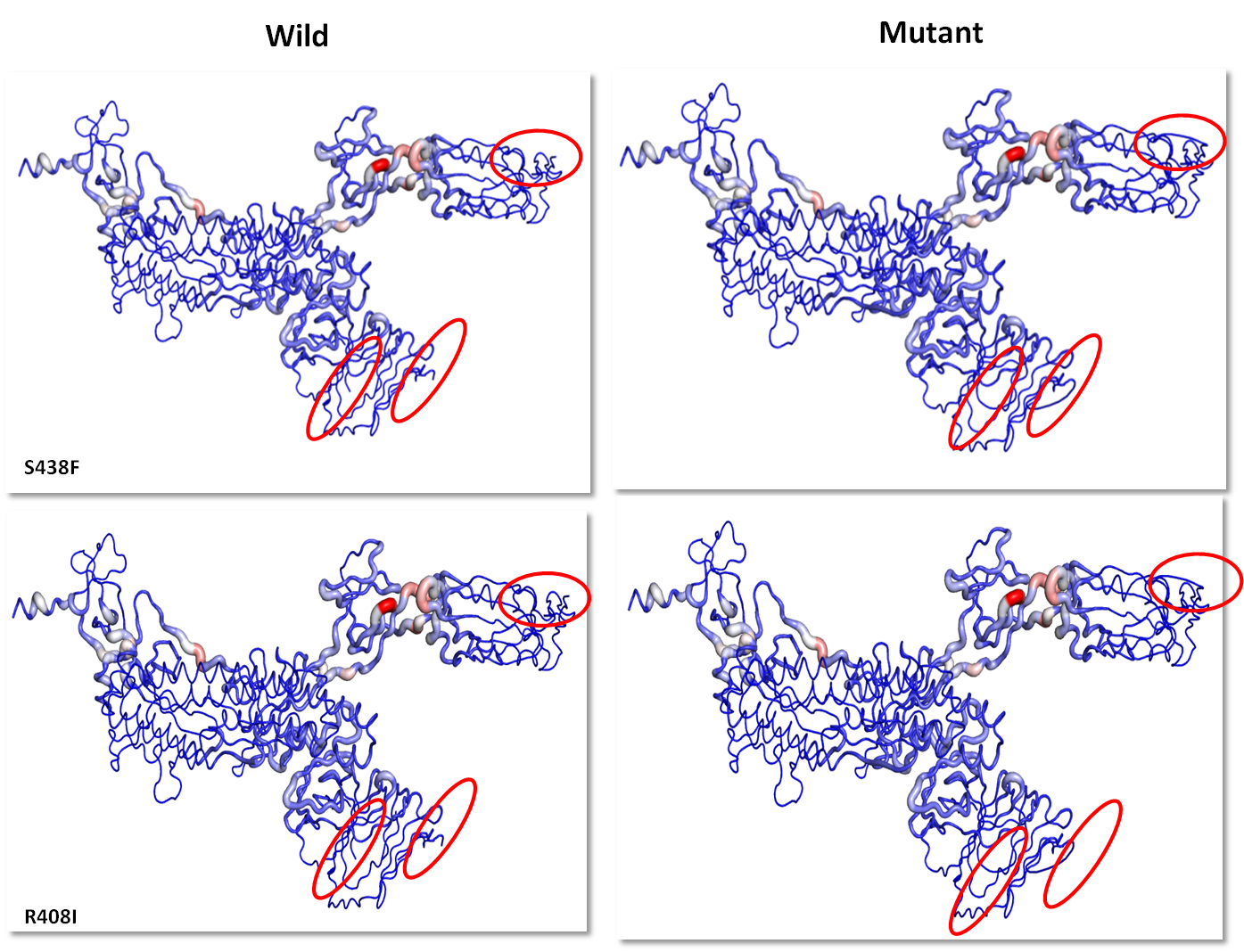


Fig. S3. Visual analysis of deformation energies of mutations S438F and R408I.


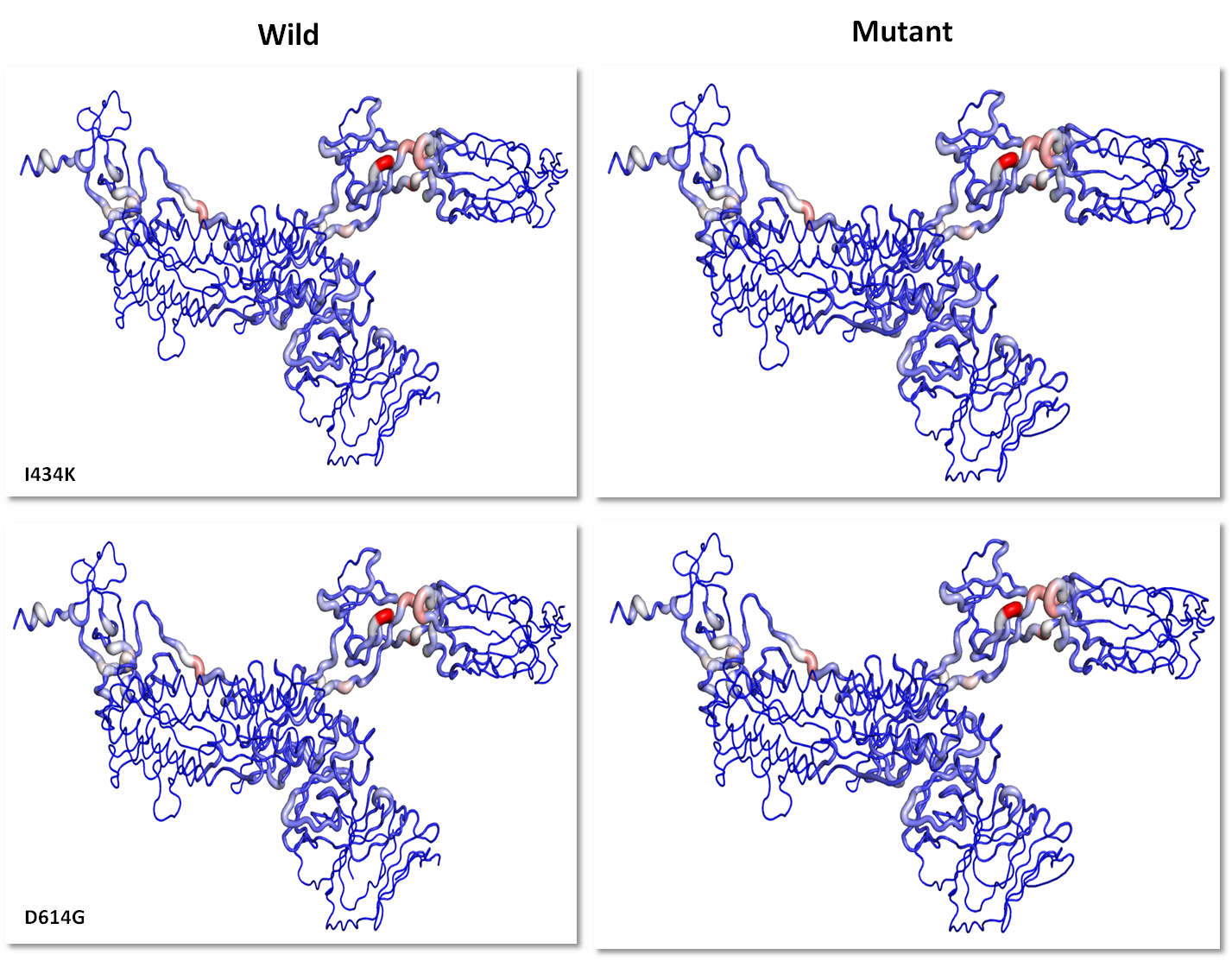


Fig. S4. Visual analysis of deformation energies of mutations I434K and D614G.


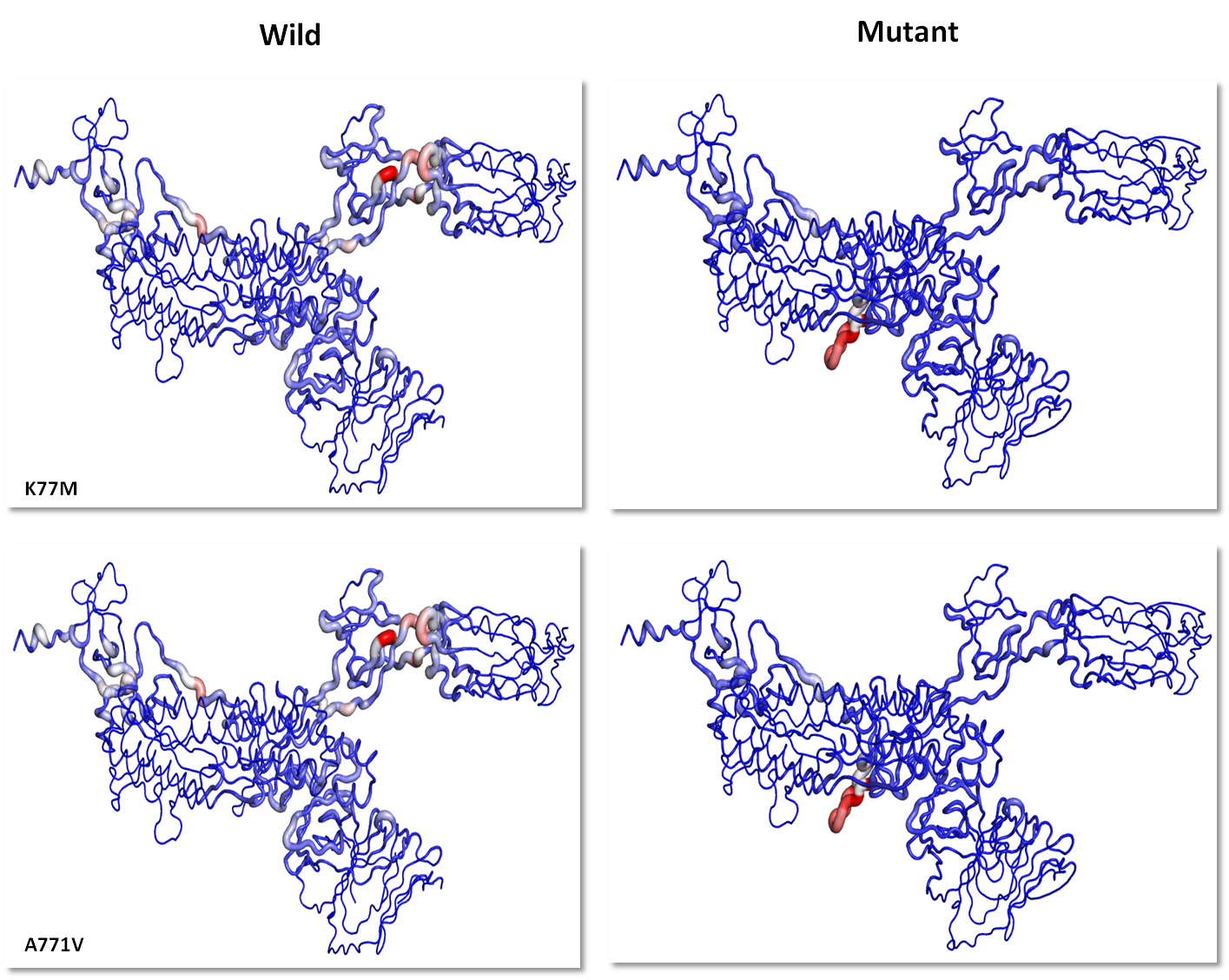


Fig. S5. Visual analysis of deformation energies of mutations K77M and A771V.
